# Affective Computing Approaches in Child–Robot Interaction: A Systematic Review and Taxonomy

**DOI:** 10.3390/s26185721

**Published:** 2026-09-09

**Authors:** Sandra Cano, Juan Pablo Vásconez, Kiara Villarroel, Juan Carlos Geraldo, Sergio Albiol-Pérez

**Affiliations:** 1 Schools of Informatics Engineering, Pontificia Universidad Católica de Valparaíso, Valparaíso 2340000, Chile; kiara.villarroel@pucv.cl (K.V.); juan.geraldo@pucv.cl (J.C.G.); 2 Faculty of Engineering, Universidad Andres Bello, Santiago 7500735, Chile; juan.vasconez@unab.cl; 3 ANID—Millennium Nucleus in Data Science for Plant Resilience (PhytoLearning), Santiago 8370251, Chile; 4 Aragón Health Research Institute (IIS Aragón), Universidad de Zaragoza, Cdad. Escolar, 4, 44003 Teruel, Spain; salbiol@unizar.es

**Keywords:** human–robot interaction, social robots, affective computing, child–robot interaction, multimodal sensing, robot expressiveness

## Abstract

Affective computing has become increasingly relevant in child–robot interaction (CRI), particularly in social robotics, emotion recognition, engagement assessment, and autism-related interventions. This systematic review with a critical and integrative synthesis analyzes 105 included studies to examine how affect is sensed, represented, processed, expressed, and evaluated in CRI. The literature search was conducted in IEEE Xplore, Web of Science, Scopus, and PubMed, following a systematic screening process guided by the review objectives. A descriptive and structured narrative synthesis was conducted considering publication characteristics, robot platform and morphology, target population, sensing modalities and observed affect-relevant features, affective constructs and representation models, computational and control mechanisms, robot affective expression, evaluation strategies, and remaining research gaps. The findings show a strong emphasis on ASD-related contexts, visually observable and behavioral features, facial emotion recognition, body movement analysis, and engagement assessment. The review also identifies important limitations, including reliance on camera-based affect recognition, comparatively limited use of physiological and other complementary sensing modalities, unclear alignment between robot roles and interaction strategies, insufficient reporting of robot emotional expressiveness and control mechanisms, and limited attention to explainability, data governance, and long-term ethical implications. Based on these findings, an integrative taxonomy of affective computing in CRI is proposed, comprising six interconnected dimensions: interaction context; sensing modalities and observed features; affective constructs and representation models; computational and control mechanisms; robot affective expression; and evaluation and adaptation strategies. Rather than treating these dimensions as entirely novel categories, the taxonomy consolidates and extends previously fragmented classifications into a child-centered representation of the affective interaction process. Overall, this review argues that affective CRI should move beyond automatic emotion recognition toward multimodal, embodied, developmentally appropriate, explainable, and ethically grounded robot interaction.

## 1. Introduction

Affective Computing (AC) was introduced by Rosalind W. Picard as a field concerned with the design of computational systems capable of recognizing, interpreting, and responding to human emotions [1]. In Human–Robot Interaction (HRI), AC has gained increasing relevance because social robots require affective capabilities to perceive users’ emotional states, generate appropriate expressive behaviors, and support more natural, adaptive, and socially meaningful interactions [2].

Within Human–Robot Interaction, social robotics has emerged as a multidisciplinary research field that integrates contributions from affective computing, Social Signal Processing (SSP), Computer Vision (CV), and Machine Learning (ML). Nevertheless, current research still lacks a clear and unified understanding of how affective sensing, perception, and interpretation methods can be designed, implemented, and evaluated in social robotic systems for children.

Child–Robot Interaction (CRI) can be understood as a subfield of HRI in which the primary users are children [3]. However, CRI cannot be treated simply as HRI with younger participants, since children’s cognitive, emotional, social, and developmental characteristics shape how they perceive, interpret, and respond to robots [4]. In particular, younger children often show high levels of initial engagement with social robots, partly because robots can be perceived as socially meaningful, animate, and emotionally expressive agents [5]. Nevertheless, such engagement should not be assumed to be automatic or sustainable over time. It may be influenced by the novelty effect, the child’s developmental stage, individual characteristics, the robot’s embodiment, and the quality of the interaction design [6]. Therefore, affective computing approaches in CRI should move beyond short-term engagement and address how robots can support emotionally coherent, adaptive, and developmentally appropriate interactions with children [7].

This distinction is particularly important for affective computing in social robotics. While short-term interaction with children may be easier to elicit than long-term engagement, sustaining meaningful child–robot interaction requires more than a basic social repertoire [8]. Robots must be able to perceive affective and behavioral cues, interpret them in relation to the child’s context, and generate expressive responses that are understandable, coherent, and appropriate for the child’s developmental level [9]. Recent studies suggest that children’s engagement with social robots is shaped by individual differences such as temperament, anxiety, and verbal participation. For example, ref. [5] found that children’s generalized anxiety was associated with lower positive emotional engagement, while surgency was related to greater verbal engagement during preschool child–robot interaction. These findings highlight the need for child-centered [10] and personalized affective interaction models [11]. This developmental perspective is also relevant for language-based interaction. Children are still developing their linguistic abilities and may produce linguistic errors during communication; at the same time, they may not always notice errors made by adult interlocutors or robots [12]. Therefore, affective and social robotic systems designed for children should consider not only emotional engagement but also developmental differences in language comprehension, error perception, and communicative expectations [9].

A key element underlying interactions with human teachers and other social agents, such as social robots, is engagement [13]. In educational contexts, engagement is commonly understood as a multidimensional construct that reflects the active commitment, participation, and involvement of students in learning activities [14]. Ref. [14] proposed that engagement consists of three interrelated dimensions: behavioral, emotional, and cognitive engagement. Behavioral engagement refers to observable actions that demonstrate a child’s participation in the learning task, such as attention to the task, persistence, participation, and compliance with classroom norms. Emotional engagement involves the child’s affective reactions, including interest, enjoyment, motivation, and positive emotional responses toward the learning activity [15]. Cognitive engagement refers to the mental investment required to understand, process, and reflect on the learning task. In CRI, these dimensions are particularly relevant because social robots can influence children’s affective and cognitive responses during educational interactions [13]. However, engagement should not be understood as a fixed property of the child or the robot, but as a dynamic process shaped by the interaction between the child’s individual characteristics, the learning environment, the task, and the robot’s social and expressive behavior [16].

Based on these considerations, this review argues that affective computing in CRI should not be reduced to emotion recognition or algorithmic classification. Instead, it should be examined as a multidisciplinary process that integrates affective sensing, emotional modeling, multimodal expression, adaptive behavior, and child-centered design. Therefore, a critical analysis is needed to clarify how current studies conceptualize, implement, and evaluate affective computing approaches in robotic interactions with children.

This article is structured as follows. Section 2 describes concepts related to the study. Section 3 describes the methodology used to conduct the critical review, including the search strategy, databases, inclusion and exclusion criteria, and data extraction process used in Child–Robot Interaction. Section 4 critically analyzes the emotional models, affective sensing methods, computational techniques, and expressive behaviors implemented in social robots for children. Section 5 discusses the main methodological, technical, theoretical, and ethical gaps identified in the literature. Finally, Section 6 presents the conclusions and outlines future research directions for the development of emotionally coherent, adaptive, and child-centered social robots.

## 2. Background

### 2.1. Human–Robot Interaction (HRI)

Human–Robot Interaction (HRI) is an interdisciplinary field that studies how humans and robots communicate, collaborate, and influence each other in shared physical, social, educational, or therapeutic environments [17]. In its early development, HRI focused mainly on usability, task execution, safety, control, and collaboration between humans and robotic systems. However, in recent years, the field has expanded significantly with the integration of artificial intelligence (AI), enabling robots to perceive user behavior, adapt their responses, and provide increasingly personalized interaction experiences [18]. This evolution has contributed to the development of socially interactive robots, where communication, engagement, trust, emotion, and social behavior have become central dimensions of the interaction [19].

From this broader field, Child–Robot Interaction (CRI) has emerged as a specialized area focused on how children perceive, interact with, learn from, and respond emotionally and socially to robots. CRI cannot be considered merely a subfield of HRI for younger users because children differ from adults in cognitive development, emotional regulation, language skills, attention, social understanding, and their tendency to attribute agency or intentionality to robotic agents [9]. Therefore, robots designed for children must consider developmental stage, playfulness, safety, accessibility, expressiveness, pedagogical goals, and ethical safeguards. In educational and therapeutic contexts, especially with children with autism spectrum disorder (ASD) [20], robots have been used as tutors, peers, mediators, or assistive agents to support learning, emotion recognition, imitation, joint attention, engagement, and social communication. CRI can be considered a child-centered derivation of HRI that integrates robotics, developmental psychology, education, therapy, social interaction, and affective computing. While general HRI asks how humans and robots interact, CRI asks how robots can interact meaningfully, safely, and adaptively with children, taking into account their developmental, emotional, social, and ethical needs.

### 2.2. Affective Computing

Affective computing refers to the design of systems capable of recognizing, interpreting, modeling, and responding to human emotions and affective states [1]. Originally proposed as a field that connects computation with human emotion, affective computing is not limited to machine learning or facial emotion recognition; rather, it includes emotion theory, cognitive modeling, signal processing, multimodal sensing, user experience, ethics, and adaptive interaction design [21]. In child–robot interaction, affective computing enables robots to detect and respond to children’s emotional and social signals, such as facial expressions, gaze, body movement, voice, behavioral engagement, proximity, physiological signals, and task performance.

Affective states can be represented through different emotional models. Some studies use discrete emotion models, classifying emotions proposed by Ekman [22] such as happiness, sadness, anger, fear, surprise, disgust, or neutral states. Other studies use dimensional models such as the circumplex model [23], Plutchik’s model [24], and PAD (pleasure–arousal–dominance) model [25], where valence and arousal are used to represent emotions as a continuous state rather than a fixed label. Additional approaches include FACS-based action units [26], PAD models, embodied robot models [27], stress/arousal indicators, empathy-related constructs, and multimodal representations that combine several affective channels [28].

Affective computing in child–robot interaction should be understood not only as the automatic recognition of emotions but also as part of a broader interdisciplinary approach to emotion. Emotions are complex phenomena that involve cognition, bodily states, action, motivation, social interaction, and the environment [29]. From an embodied perspective, robots can serve as models for exploring how affective processes are generated, expressed, regulated, and used to guide behavior. This view is particularly relevant for CRI because children’s emotions are not only facial displays to be classified, but dynamic states that emerge through interaction, movement, attention, regulation, and context.

In the context of CRI, multimodal signal processing provides a foundation for affective sensing and interpretation. Ref. [30] describes multimodal signal processing in human–robot interaction as the integration of several computational approaches, including computer vision, natural language processing, gesture recognition, and emotion recognition. These modalities are captured through intelligent sensors and processed to enable robots to perceive, interpret, and respond to human behavior in more adaptive and context-aware ways.

### 2.3. Positioning Against Previous Reviews

Previous reviews have addressed several components relevant to affective Child–Robot Interaction, although from different analytical perspectives. Existing work has examined affective computing in Human–Robot Interaction, affective techniques in infant-robot interaction, affective communication in socially assistive robots for children with ASD, and children’s engagement in digital and robotic activities. These contributions provide important foundations for understanding affect recognition, multimodal sensing, engagement, emotional communication, and adaptive interaction. However, these aspects have generally been examined separately or within more specific populations, interaction goals, or analytical perspectives. The present review therefore does not assume that each individual component of the proposed taxonomy is novel. Instead, its contribution lies in integrating these previously fragmented aspects into a single CRI-specific framework and in defining explicit boundaries among sensing modalities, observed features, affective constructs, computational and control mechanisms, robot affective expression, and evaluation/adaptation.

As shown in Table 1, the proposed taxonomy combines consolidation and extension rather than introducing six entirely new categories. Interaction context and affective constructs consolidate dimensions already addressed in previous CRI and affective-robotics research. The sensing dimension extends previous recognition-oriented classifications by explicitly separating sensing modalities or data sources from the observable features derived from them. Computational and control mechanisms extend the analysis from affect inference to the mechanisms through which affective information influences interaction control. Robot affective expression consolidates the output side of affective communication as an explicit functional dimension, while evaluation and adaptation connect affect perception and expression to interaction outcomes and behavioral adjustment. The distinctive contribution of the taxonomy is therefore the integration of these components into a single child-centered affective interaction framework with explicit operational boundaries.

## 3. Methodology

This study was conducted as a systematic review with a critical and integrative synthesis and was reported in accordance with the PRISMA 2020 statement [31]. The review process comprised identification, screening, eligibility assessment, data extraction, and synthesis. Systematic searches were conducted in IEEE Xplore, Web of Science, PubMed, and Scopus for studies published from January 2014 to 15 June 2026. The review protocol was registered in PROSPERO (CRD420261466786). The terms “critical” and “integrative” refer to the synthesis and interpretation of the included evidence and do not indicate a separate hybrid review design.

Eligible studies involved children as participants or direct target users; included a physical robot in a social, educational, therapeutic, or assistive interaction; and addressed at least one affective, emotional, engagement, or socio-emotional component. Study selection and data extraction were performed using predefined eligibility and coding criteria.

Because substantial heterogeneity was observed across study populations, robot platforms, research designs, interaction contexts, affective measures, computational techniques, and reported outcomes, a meta-analysis was not considered appropriate. Therefore, descriptive statistical and structured narrative syntheses were conducted.

### 3.1. Research Design

The review adopted a critical and integrative approach. Rather than focusing only on the technical performance of emotion recognition algorithms, the analysis examined how affective computing is conceptualized, implemented, and evaluated in child–robot interaction. Therefore, the review considered four complementary dimensions: emotional perception, emotional modeling, robot expressive behavior, and adaptive interaction.

The aim was not only to identify the technologies used in previous studies but also to examine their theoretical, methodological, and ethical foundations. This perspective is particularly relevant because affective computing in child–robot interaction involves more than machine learning; it also includes psychological models of emotion, multimodal communication, embodiment, child-centered design, and the social interpretation of robotic behavior.

### 3.2. Research Questions

The review was guided by the following research questions:

- RQ1: What affective computing approaches have been used in Child–Robot Interaction?
- RQ2: What sensing modalities and observable behavioral or physiological features are used to capture affect-relevant information from children during interactions with social robots?
- RQ3: What affective constructs and representation models are used to represent or infer children’s affective states?
- RQ4: What computational techniques are applied to support affective sensing, perception, and understanding in child–robot interactions?
- RQ5: How do social robots express emotions or affective states during interactions with children?
- RQ6: How are engagement, emotional response, acceptance, or adaptive behavior evaluated in affective Child–Robot Interaction studies?
- RQ7: What methodological, technical, theoretical, and ethical gaps remain in the development of affective computing approaches for social robotics with children?

### 3.3. Search Strategy

As shown in Table 2, the search strategy was structured using three conceptual blocks combined through Boolean operators. The first block included terms related to affective computing and emotional processing; the second block included terms associated with child–robot interaction and social, educational, or assistive robotics; and the third block focused on child populations, including children with autism spectrum disorder. Formally, the search equation can be represented as follows:

S = A AND R AND C

### 3.4. Inclusion and Exclusion Criteria

Studies were included if they met the following criteria:

1. The study involved children as participants or target users.
2. The study addressed child–robot interaction, human–robot interaction with children, educational robotics, social robotics, or socially assistive robotics.
3. The study included an affective or emotional component, such as affective computing, emotion recognition, emotion detection, emotion expression, emotional adaptation, engagement, empathy, emotional response, or socio-emotional interaction.
4. The study used a physical robot as part of the interaction. Studies based only on virtual agents, avatars, or chatbots were not considered.
5. The study described at least one relevant methodological, computational, or interaction-design element, such as sensors, emotional models, affective signals, machine learning methods, robot behaviors, or evaluation instruments.
6. The study was published between 2014 and 2026.
7. The study was published in English as a peer-reviewed journal article or conference paper.

Studies were excluded if they met any of the following criteria:

1. The study focused only on adults or did not clearly involve children as users or participants.
2. The study did not include a physical robot.
3. The study focused only on general robotics, navigation, mechanical design, or programming without an affective, emotional, or socio-emotional component.
4. The study addressed education, learning, or self-regulated learning without a clear connection to affective computing or child–robot interaction.
5. The study used only virtual agents, conversational agents, screen-based avatars, or chatbots.
6. The study did not provide sufficient methodological or technical information to support data extraction.
7. The study was not written in English.
8. The publication was a thesis, book chapter, editorial, abstract, poster, short summary, or non-peer-reviewed document.
9. Systematic literature reviews, scoping reviews, and narrative reviews were excluded from the main empirical synthesis, although they were used as background sources to contextualize the field and identify relevant concepts, gaps, and terminology.

### 3.5. Study Selection

After applying the inclusion and exclusion criteria, the selected records were screened in two stages. First, titles and abstracts were reviewed to identify studies related to affective computing in Child–Robot Interaction. Second, the full texts of potentially relevant studies were examined to confirm their eligibility. Duplicate records and studies without a clear affective, emotional, or child–robot interaction component were removed.

The included studies were then analyzed through a critical appraisal process focused on five dimensions: theoretical grounding, technical implementation, child-centered design, methodological quality, and ethical considerations.

Theoretical grounding examined whether the affective or emotional construct was explicitly defined and whether the selected representation model (e.g., discrete emotions, valence–arousal, PAD, engagement, or related constructs) was theoretically motivated. Technical implementation examined whether the sensing modalities, observed features, computational methods, and robot expressive or adaptive mechanisms were sufficiently described to understand the affective-processing approach. Child-centered design considered whether developmental stage, individual differences, communication abilities, interaction needs, and other child-specific characteristics were addressed. Methodological quality considered the study design, sample characteristics, interaction context and duration, evaluation procedures, and ecological validity when these were reported. Ethical considerations examined the reporting of ethics approval, informed consent or assent, privacy and data protection, transparency, and emotional safety.

This critical appraisal made it possible to identify not only the main affective computing approaches used in child–robot interaction, but also their conceptual, technical, methodological, and ethical limitations.

### 3.6. Data Synthesis

A mixed descriptive and structured narrative synthesis was conducted across the final corpus of 105 included studies. Quantitative summaries were generated for the standardized variables extracted from each article, including country, publication year, article type, robot platform and morphology, target population, affective input modality, emotional or social construct, computational method, and keywords. The five-dimensional critical appraisal described in Section 3.5 was used to support the interpretation of recurrent strengths, limitations, and research gaps across the corpus.

The final statistical dataset comprised 105 included articles (see Appendix A.2). For each article, the following variables were extracted and standardized: country of study, year of publication, article type, robot platform, robot morphology, target population, sensing modality/data source and observed affect-relevant feature or behavior, emotional or social construct, computational method, and keywords.

**Figure 1:**
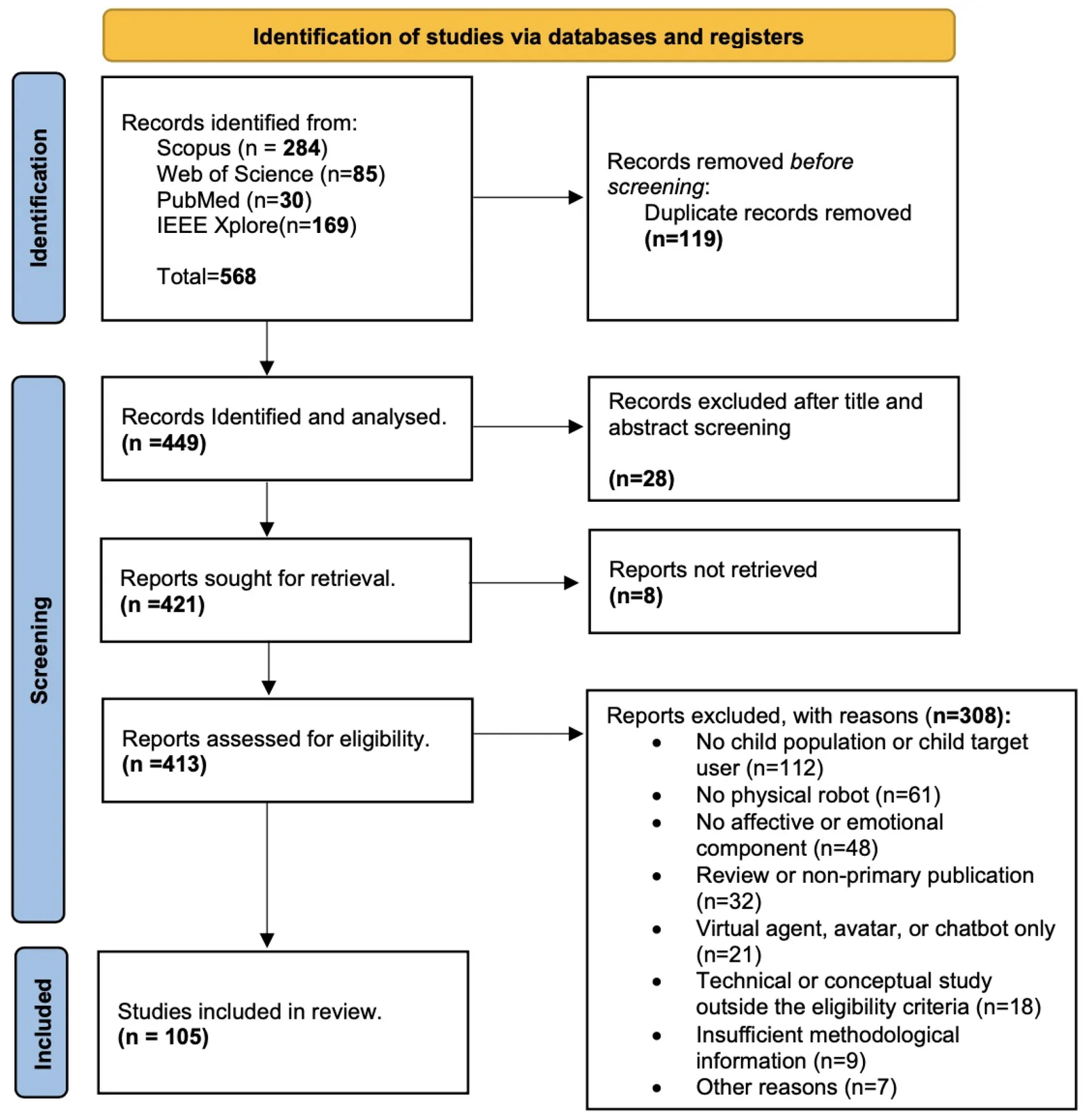
PRISMA 2020 flow diagram of the study identification, screening, eligibility assessment, and inclusion process.

Figure 1 presents the PRISMA 2020 study-selection process. The completed PRISMA 2020 checklist is provided in the Supplementary Material. The database searches identified 568 records. After removing 119 duplicates, 449 records were screened by title and abstract. Twenty-eight records were excluded during this stage. Of the 421 reports sought for retrieval, eight could not be retrieved. A total of 413 full-text reports were assessed for eligibility, of which 308 were excluded for the reasons reported in Figure 1, resulting in 105 studies included in the final synthesis.

To avoid conceptual overlap between sensing, observed behavior, affective interpretation, and computational processing, the extracted information was organized into four distinct analytical levels: sensing modality/data source, observed feature or behavior, inferred affective construct, and computational method. Table 3 summarizes the relationships among the first three levels, whereas computational methods are analyzed separately in RQ4. This distinction was subsequently maintained throughout the analysis of RQ2–RQ4 and in the construction of the proposed taxonomy.

Missing and non-classifiable information was handled using predefined coding rules. “Not reported (NR)” was assigned when the original article did not provide sufficient information to determine a variable. “Not applicable (NA)” was used when a variable was not relevant to the study design or analytical approach. “Other” was assigned when the information was explicitly reported but did not fit any of the predefined categories.

## 4. Results

### 4.1. Data Extraction and Synthesis

**Figure 2:**
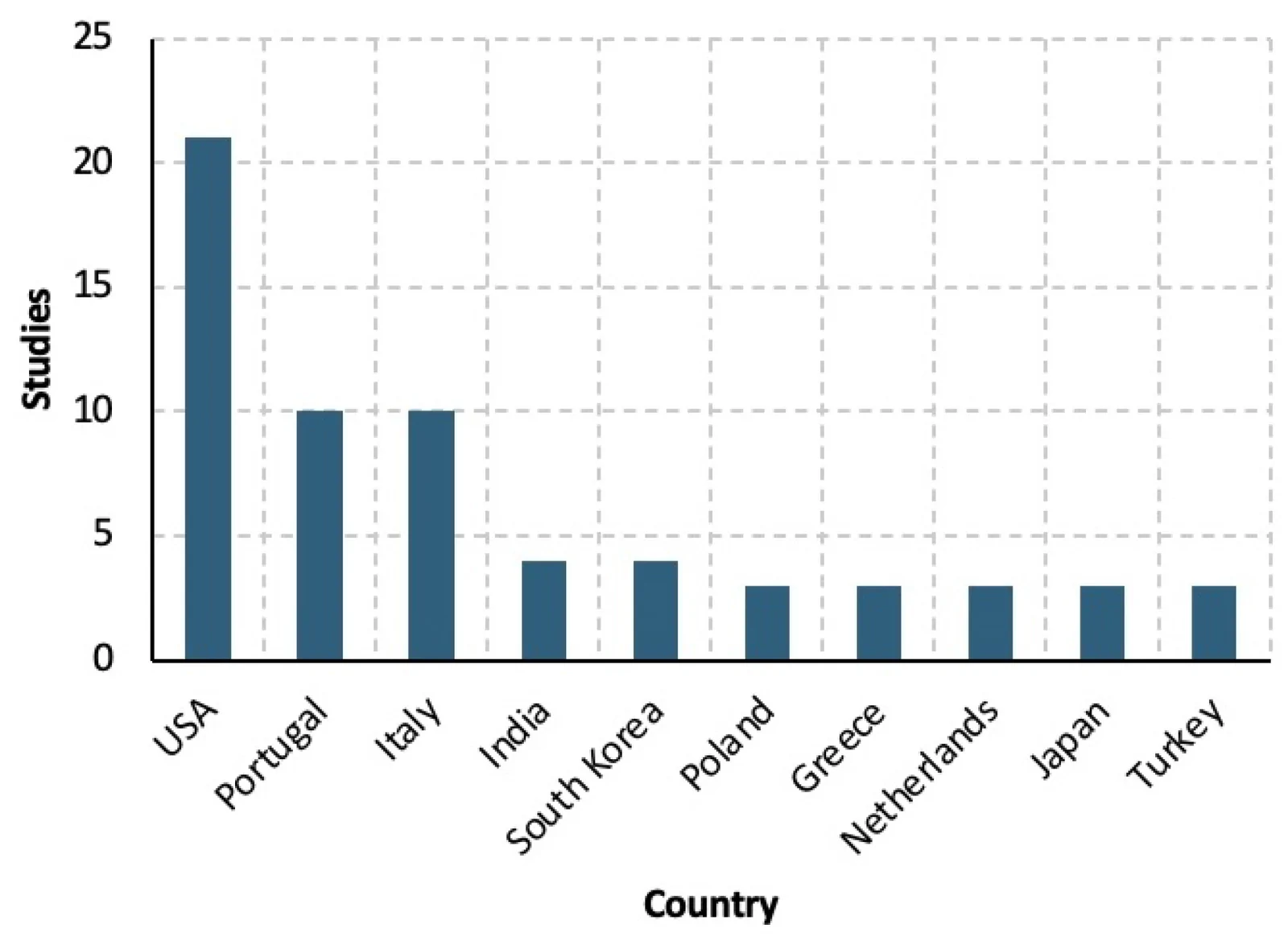
Distribution of studies by country.

Figure 2 shows that the geographical distribution of studies is concentrated in a limited number of countries, with the United States representing the largest contribution, accounting for 21 studies (20.0%) of the included corpus. Portugal and Italy followed, each with 10 studies (9.5%), indicating a strong European contribution to the field. Other countries with notable participation included India and South Korea, with four studies each (3.8%), as well as Poland, Greece, the Netherlands, Japan, and Turkey, each contributing three studies (2.9%). Overall, the findings suggest that research on child–robot interaction, autism, social robotics, and affective computing is led mainly by North America and Europe, while also showing increasing participation from Asian and other international research contexts.

**Figure 3:**
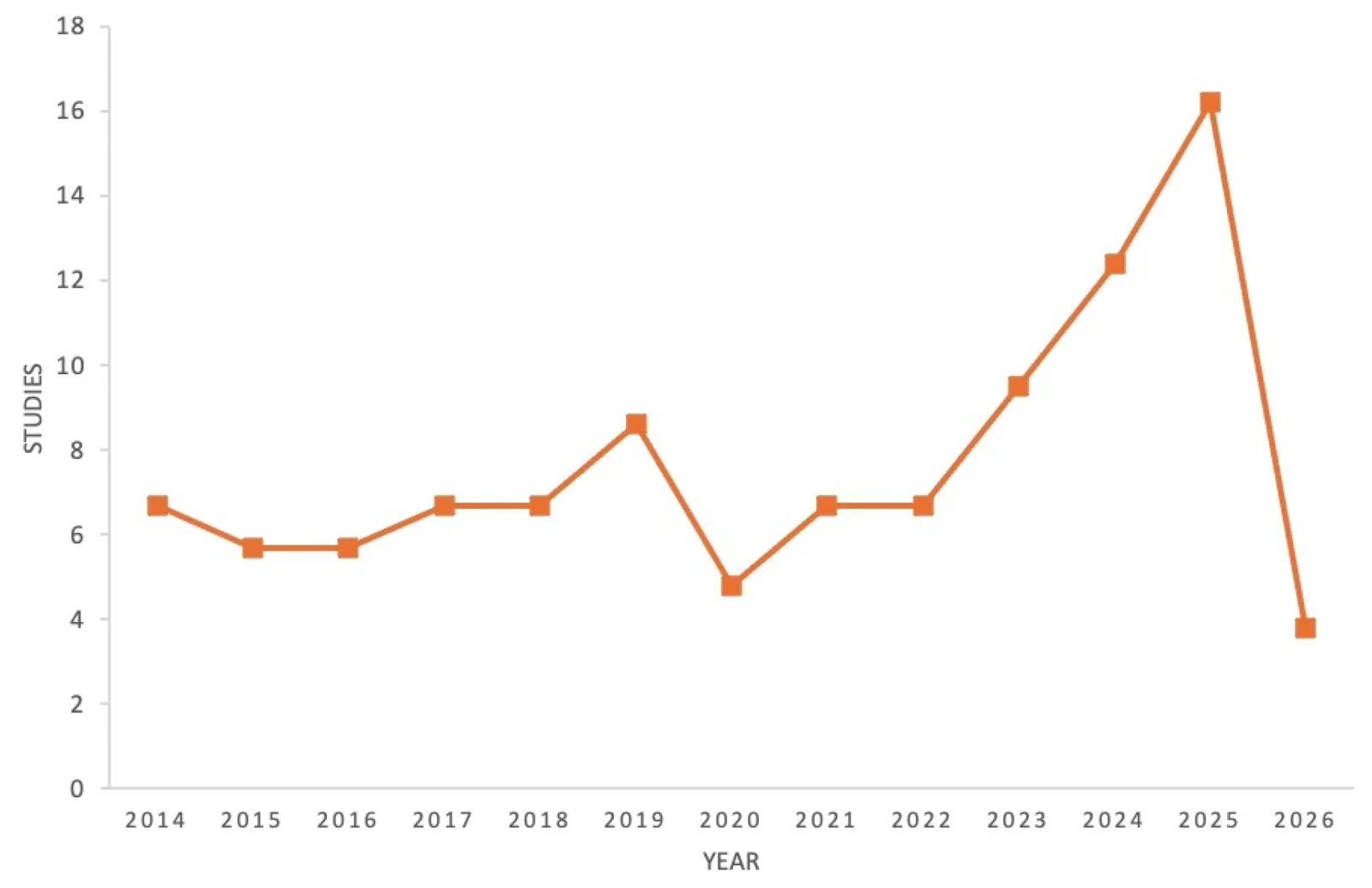
Distribution of studies by year.

Figure 3 shows the distribution of studies by publication year. The number of publications remained relatively stable between 2014 and 2022, ranging from 5 to 9 studies per year. However, the highest concentration of studies was observed in the most recent period, especially between 2023 and 2025, which together accounted for 40 studies, representing 38.1% of the included corpus. The peak occurred in 2025, with 17 studies (16.2%), followed by 2024 with 13 studies (12.4%) and 2023 with 10 studies (9.5%). This pattern suggests a growing research interest in recent years, particularly in the use of social robots, child–robot interaction, autism-related interventions, and affective computing approaches. The lower number observed in 2026 (4 studies; 3.8%) may reflect the fact that the year is still emerging within the analyzed dataset.

**Figure 4:**
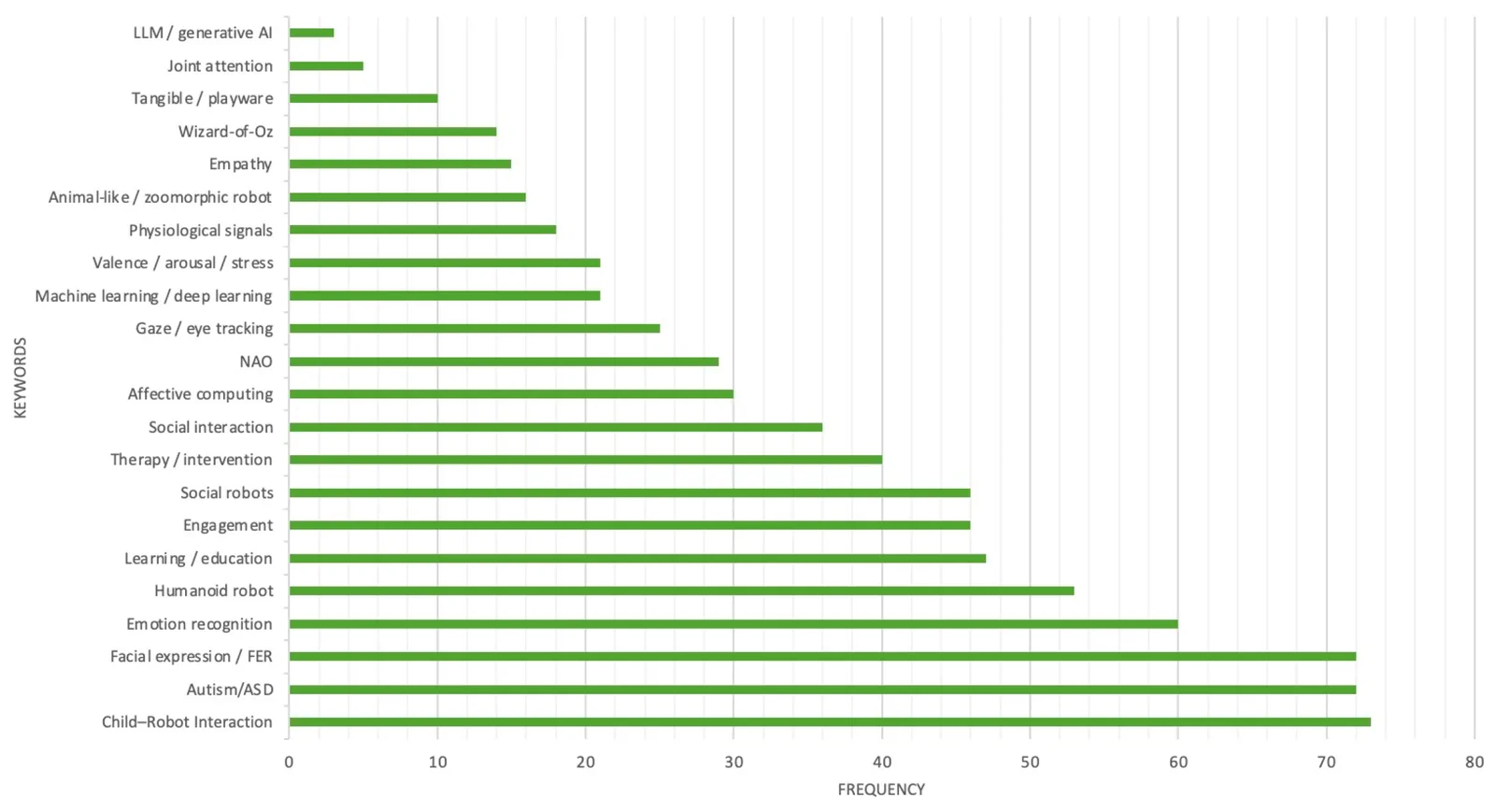
Frequency of recurrent thematic keywords/topics coded in the included studies.

Figure 4 shows the frequency of thematic keywords/topics coded across the included studies. The literature was mainly concentrated around Child–Robot Interaction and Autism/ASD, which were the most frequent topics, appearing in 73 and 72 studies, respectively. A strong emphasis was also observed on Facial expression/FER (n=72) and Emotion recognition (n=60), indicating the central role of visual affective cues and emotional classification in the field. Within the thematic keyword/topic coding. Humanoid robot appeared in 53 studies and Social robot in 46 studies, reflecting the prominence of socially interactive and human-like robotic systems. Educational and therapeutic orientations were also prominent, with Learning/education identified in 47 studies and Therapy/intervention in 40. Other recurrent topics included Engagement (n=46), Social interaction (n=36), and Affective computing (n=30). Less frequent but emerging themes included physiological signals, animal-like or zoomorphic robots, empathy, Wizard-of-Oz approaches, tangible/playware interfaces, joint attention, and LLM/generative AI, suggesting increasing diversification in the methods and technologies used in child–robot interaction research.

**Figure 5:**
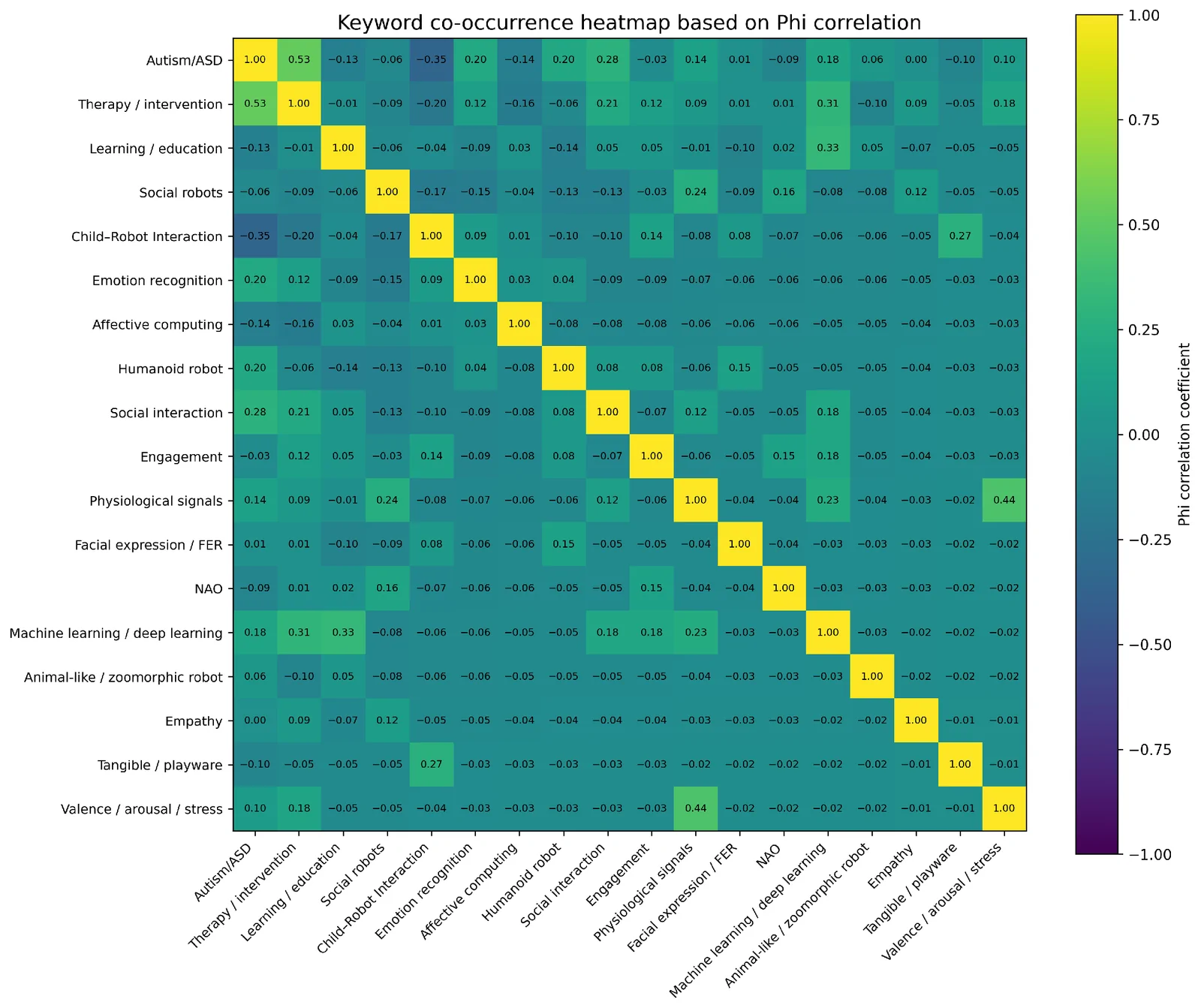
Phi-correlation heatmap among the 18 recurrent thematic keywords/topics coded in the included studies.

Figure 5 presents the pairwise Phi correlations among the 18 recurrent thematic keywords/topics coded as binary variables in the included studies. Positive Phi values indicate that two topics tended to co-occur more frequently than expected from their marginal frequencies, values close to zero indicate weak or negligible association, and negative values indicate an inverse association. The strongest positive association was observed between Autism/ASD and Therapy/intervention (Phi = 0.52), reflecting the strong therapeutic orientation of autism-related research in the corpus. A second notable association was found between Physiological signals and Valence/arousal/stress (Phi = 0.44), indicating that physiological sensing was particularly associated with dimensional or arousal-related affective constructs. Other moderate positive associations included Physiological signals with Machine learning/deep learning (Phi = 0.30), Autism/ASD with Social interaction (Phi = 0.28), Learning/education with Machine learning/deep learning (Phi = 0.28), and Child–Robot Interaction with Tangible/playware approaches (Phi = 0.27). Most remaining pairwise associations were weak, suggesting that the thematic structure of affective Child–Robot Interaction is distributed across several relatively distinct research strands rather than being dominated by a single cluster of strongly co-occurring topics.

**Figure 6:**
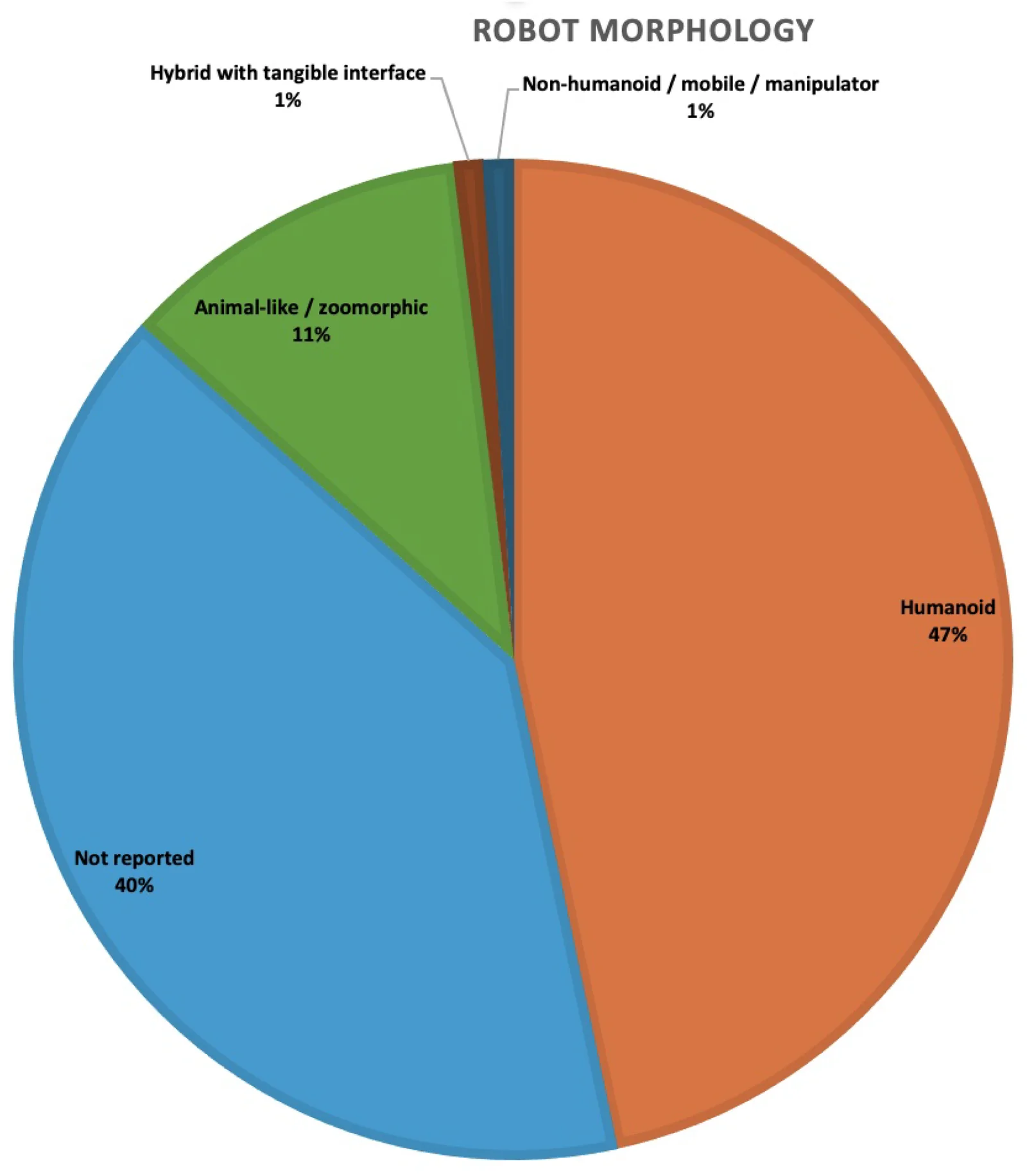
Robot morphology.

Figure 6 presents the standardized robot-morphology classification across the 105 included studies. Humanoid morphology was explicitly identified in 49 studies (46.7%). Robot morphology was not reported or could not be determined with sufficient confidence in 42 studies (40.0%). Importantly, the “not reported” category refers only to insufficient information for assigning a standardized morphology and should not be interpreted as indicating the absence of a physical robot. Studies based exclusively on virtual agents, avatars, or chatbots were excluded according to the eligibility criteria. Animal-like or zoomorphic robots were identified in 12 studies (11.4%), while hybrid tangible interfaces and non-humanoid/mobile/manipulator robots were each identified in one study (1.0%).

Social robots played several roles in child–robot interaction rather than functioning only as technological intermediaries. Their most frequent role was that of an affective and social interaction partner, supporting emotion recognition, engagement, imitation, turn-taking, and social communication. Robots also acted as educational tutors or learning companions, particularly in studies focused on social–emotional learning, storytelling, emotional vocabulary, theory of mind, and cognitive or socio-emotional skills, a role identified in 45 studies (42.9%). In autism-related contexts, robots frequently functioned as therapeutic or intervention support agents, appearing in 40 studies (38.1%), where they helped structure tasks, provide predictable cues, reinforce behaviors, and support therapist-guided activities.

In addition, robots served as engagement facilitators in 46 studies (43.8%), using gestures, speech, movement, feedback, and expressive behaviors to sustain children’s attention and participation. Finally, in some studies, robots also acted as affective sensing platforms, enabling researchers to capture children’s facial expressions, gaze, body movement, task performance, voice, or physiological responses during interaction. Therefore, the role of the robot in CRI can be understood as multidimensional: robots operate as tutors, therapeutic assistants, social mediators, affective companions, and data-elicitation platforms, depending on the goal of the interaction and the child population involved.

Regarding the specific robotic platforms used in the included studies, NAO was the most frequently employed robot, appearing in 29 studies (27.6%), followed by ZECA/Zeno R50, which appeared in 8 studies (7.6%). This suggests that affective child–robot interaction research has relied predominantly on well-established humanoid platforms, particularly NAO, likely due to its availability, programmability, and capacity to support gestures, speech, posture, LED-based expressions, and educational or therapeutic interaction scenarios. Other platforms, such as Kaspar and Pepper, were less frequent, indicating that the field remains technologically concentrated around a small number of widely used social robot platforms.

The qualitative critical appraisal revealed recurrent limitations across the five dimensions considered. Theoretical grounding was frequently centered on discrete emotion categories or engagement constructs, with less frequent use of dimensional or developmentally grounded affective models. Technical implementation was generally described in studies using sensing and computational methods, although the mechanisms linking affect recognition to robot adaptation were often insufficiently specified. Child-centered considerations were more explicit in ASD-related interventions, but developmental and individual differences were not consistently incorporated into computational models. Methodological limitations included small samples, short-term interactions, heterogeneous evaluation protocols, and limited ecological validation. Ethical reporting commonly addressed informed consent or ethics approval, whereas privacy, data governance, explainability, long-term data retention, and transparency were less consistently described.

### 4.2. Response to Research Questions

#### 4.2.1. RQ1: What Affective Computing Approaches Have Been Used in Child–Robot Interaction?

Affective computing in child–robot interaction was mainly implemented through multimodal affect detection, emotion recognition, engagement assessment, physiological sensing, gaze analysis, and adaptive robot expression. Since several studies used more than one approach, the categories are not mutually exclusive. The most frequent approach was the analysis of body posture, gestures, and expressive movement, identified in 72 studies (68.6%), followed by facial expression recognition/FER, present in 68 studies (64.8%). These approaches were commonly used to infer children’s emotional states, recognize affective expressions, or evaluate how children interpreted emotions expressed by robots, especially in humanoid platforms such as NAO, ZECA/Zeno, Kaspar, and Tega [32,33,34,35].

A second group of approaches focused on engagement and behavioral indicators, using task performance, game interaction, response time, gaze behavior, prompts, and participation patterns. Game or task-based performance indicators appeared in 37 studies (35.2%), while gaze and eye-tracking measures appeared in 24 studies (22.9%). These approaches were especially relevant in studies addressing attention, joint attention, engagement, and social communication in children with autism, where looking behavior and response to robot cues were used as indicators of interaction quality [36,37,38,39].

Several studies also incorporated voice/audio-based affective interaction, identified in 23 studies (21.9%). These approaches included verbal interaction, prosodic cues, speech-based engagement, and affective dialogue with social robots. In parallel, physiological sensing was used in 18 studies (17.1%) through signals such as heart rate, EDA, BVP, ECG, or wearable-based indicators of arousal and stress. These studies expanded affective computing beyond visible behavior by estimating internal emotional states, particularly stress, arousal, attention, and emotional regulation during robot-assisted interaction [40,41,42].

Regarding computational techniques, machine learning and deep learning were identified in 21 studies (20.0%). These methods were used for emotion recognition, engagement classification, multimodal fusion, personalized affect modeling, and automatic interpretation of behavioral or physiological signals. Examples include the use of deep learning for facial and body-based emotion recognition, personalized machine learning for affect and engagement estimation, and multimodal frameworks combining face, posture, gaze, and physiological data [11,32,43]. Statistical analysis was also common, appearing in 32 studies (30.5%), mainly to compare children’s responses, evaluate engagement, or examine relationships between affective variables and interaction outcomes.

Less frequent but emerging approaches included Wizard-of-Oz interaction designs in 14 studies (13.3%), tangible or playware-based affective interaction in 9 studies (8.6%), and LLM/generative AI approaches in 3 studies (2.9%). These newer approaches suggest a gradual shift from simple emotion detection toward more adaptive, interactive, and personalized affective systems. Overall, the results indicate that affective computing in child–robot interaction is dominated by visual and behavioral approaches, especially facial expression, gesture, engagement, and gaze analysis, while physiological sensing and generative AI remain less frequent but increasingly relevant for future adaptive and emotion-aware robotic interventions.

#### 4.2.2. RQ2: What Sensing Modalities and Observable Behavioral or Physiological Features Are Used to Capture Affect-Relevant Information from Children During Interactions with Social Robots?

Affect-relevant information from children was derived predominantly from observable visual and behavioral features. The most frequently reported features were body movement, gesture, and posture, identified in 72 studies (68.6%), followed by facial expressions and facial features, reported in 68 studies (64.8%). These observable features were commonly used to support the interpretation of children’s emotional states, engagement, imitation, and social responsiveness during child–robot interaction, particularly in autism-related contexts and humanoid robot interventions [34,35,36]. Task- and interaction-related behaviors were also frequently considered, including response accuracy, response time, prompts, help requests, task persistence, and willingness to continue the activity, and they were used as indicators of engagement and participation during robot-mediated tasks [39,44]. Gaze-related behavior constituted another relevant source of affective and social information, particularly in studies addressing attention, joint attention, and social communication, where gaze direction, fixation duration, and eye contact were used to characterize the quality of the child’s interaction with the robot or task elements [36,45]. Vocal- and speech-related features, including verbal responses, prosody, and interaction patterns, were less frequently reported but provided complementary information during robot-assisted activities [43]. Physiological features, including EDA, BVP, heart rate, HRV, and related measures, were used in a smaller group of studies to characterize internal affect-related processes such as arousal, stress, attention, and emotional regulation [40,41,46]. Overall, the findings indicate that affect-related assessment in child–robot interaction remains dominated by externally observable visual and behavioral features, particularly body movement and facial information, while vocal and physiological features are less frequently incorporated. The sensing modalities and data sources used to acquire these features are distinguished separately from the observed features themselves, while the affective constructs inferred from them and the computational methods used for their interpretation are analyzed in RQ3 and RQ4, respectively.

Table 4 summarizes the frequency of observed affect-relevant features and behaviors identified across the 105 included studies. The corresponding sensing modalities or data sources and their relationship to typical inferred affective constructs are defined separately in Table 3. To avoid conflating different stages of affective processing, the quantitative frequencies reported here refer to the observed features and behaviors identified in the corpus, whereas sensing modalities/data sources and computational methods are distinguished separately. The most frequently reported observable features were body movement, gestures, and posture (n=72; 68.6%), followed by facial expressions and facial features (n=68; 64.8%). Task and interaction-related behaviors were also common (n=37; 35.2%), including response accuracy, response time, prompts, help requests, task persistence, and participation patterns. Gaze-related behavior was identified in 24 studies (22.9%), while vocal or speech-related features appeared in 23 studies (21.9%). Thermal information was reported in 20 studies (19.0%), and physiological features such as EDA, BVP, heart rate, HRV, ECG, or related measures appeared in 18 studies (17.1%). Touch or tangible-interaction behaviors were less frequent (n=9; 8.6%). Because individual studies could incorporate multiple sources and features, these categories were not mutually exclusive. Overall, the findings indicate that affective assessment in Child–Robot Interaction remains dominated by externally observable visual and behavioral information, particularly body and facial features, whereas physiological, thermal, and touch-related information is less frequently incorporated.

#### 4.2.3. RQ3: What Affective Constructs and Representation Models Are Used to Represent or Infer Children’s Affective States?

Affect was represented mainly through discrete emotion models, especially emotion categories such as happiness, sadness, anger, fear, surprise, disgust, and neutral states. This approach appeared in 60 studies (57.1%) and was commonly used in tasks involving facial expression recognition, emotion imitation, storytelling, or robot-mediated emotion recognition activities [32,35,39]. A second relevant group of studies represented affect through engagement, attention, and social participation, identified in 47 studies (44.8%). In these cases, affect was not always modeled as a specific emotion, but as the child’s level of engagement, attention, gaze behavior, or responsiveness during the interaction [36,45].

Table 5 summarizes the affective constructs and representation models identified in the included studies. Dimensional models based on valence, arousal, or stress were less frequent, appearing in 21 studies (20.0%), but they were important in studies using physiological signals, multimodal sensing, or personalized affect recognition. These models allowed researchers to represent affect as a continuous state rather than as fixed emotion labels, especially in studies focused on arousal, stress, regulation, or engagement during autism-related robot interventions [11,34,40,43]. PAD models were not clearly represented in the 105 core included studies, suggesting that Pleasure–Arousal–Dominance has been less central than discrete emotions and valence–arousal models in this corpus.

Overall, the results indicate that the field is still dominated by discrete emotion recognition models, especially those based on facial expressions and basic emotional categories. However, there is a growing movement toward dimensional and multimodal affective models, particularly those combining facial expressions, body movement, gaze, behavior, and physiological signals to represent children’s affective states more dynamically. This shift is especially relevant for child–robot interaction and autism-related interventions, where affect may be expressed through multiple channels rather than through facial expressions alone.

#### 4.2.4. RQ4: What Computational Techniques Are Applied to Support Affective Sensing, Perception, and Understanding in Child–Robot Interactions?

Computational techniques were mainly used to support three functions: affective sensing of children’s signals, perception/classification of emotional or social states, and understanding/adaptation of the interaction. The most frequently identified techniques were machine learning and deep learning, reported in 21 studies (20.0%). These methods were applied to tasks such as facial emotion recognition, engagement detection, body-movement analysis, stress recognition, and multimodal affect classification. Common techniques included CNN-based facial expression recognition, support vector machines, XGBoost, OpenFace-based feature extraction, pose estimation, and supervised classifiers trained on facial, postural, gaze, audio, or physiological data [34,36,40,43].

Rule-based, scripted, and semi-autonomous robot behaviors were also recurrently identified in the qualitative synthesis, although these control characteristics were not systematically coded as a separate quantitative variable across the complete corpus. In these systems, the robot followed predefined interaction flows, emotional expressions, prompts, gestures, feedback rules, or storytelling sequences. This approach was common in intervention studies using robots such as NAO, Kaspar, ZECA/Zeno, and KiliRo, where the robot supported emotion recognition, imitation, turn-taking, social interaction, or therapeutic activities through programmed behaviors [35,39,44].

Wizard-of-Oz and human-in-the-loop techniques were identified in 14 studies (13.3%). These approaches allowed researchers or therapists to partially control the robot while maintaining the appearance of autonomous interaction. They were particularly useful in early-stage prototypes, pilot studies, and studies with children with ASD, where safety, personalization, and flexibility were important. However, they also show that full autonomy in affect-aware child–robot interaction remains limited.

Several studies used multimodal and hybrid architectures, combining different sensing channels such as facial expression, body posture, gaze, speech, task behavior, thermal data, and physiological signals. These architectures were important because children’s affective states, especially in ASD contexts, may not be reliably inferred from a single modality. For example, multimodal systems combined cameras, depth sensors, OpenFace/OpenPose features, audio processing, gaze tracking, wearable sensors, and behavioral logs to estimate engagement, stress, attention, or valence–arousal states [36,38,43].

Physiological signal processing was another relevant computational approach. Studies using EDA, BVP, heart rate, HRV, ECG, EEG, or wearable devices applied feature extraction, baseline comparison, threshold-based analysis, and signal-processing tools such as HeartPy, cvxEDA, and EDA Explorer to infer arousal, stress, or emotional regulation [40,46]. These methods contributed to affective understanding beyond visible behavior, although they appeared less frequently than visual techniques.

Emerging approaches such as LLM/generative AI appeared in only three studies (2.9%), suggesting that generative AI is still in an early stage within child–robot affective interaction. Similarly, reinforcement learning and fuzzy logic were rare or not dominant in the 105 included empirical studies. Overall, the evidence shows that the field is currently dominated by supervised machine learning, deep learning, computer vision, scripted robot behaviors, Wizard-of-Oz control, and hybrid multimodal architectures, while more autonomous and adaptive computational models remain underexplored.

**Figure 7:**
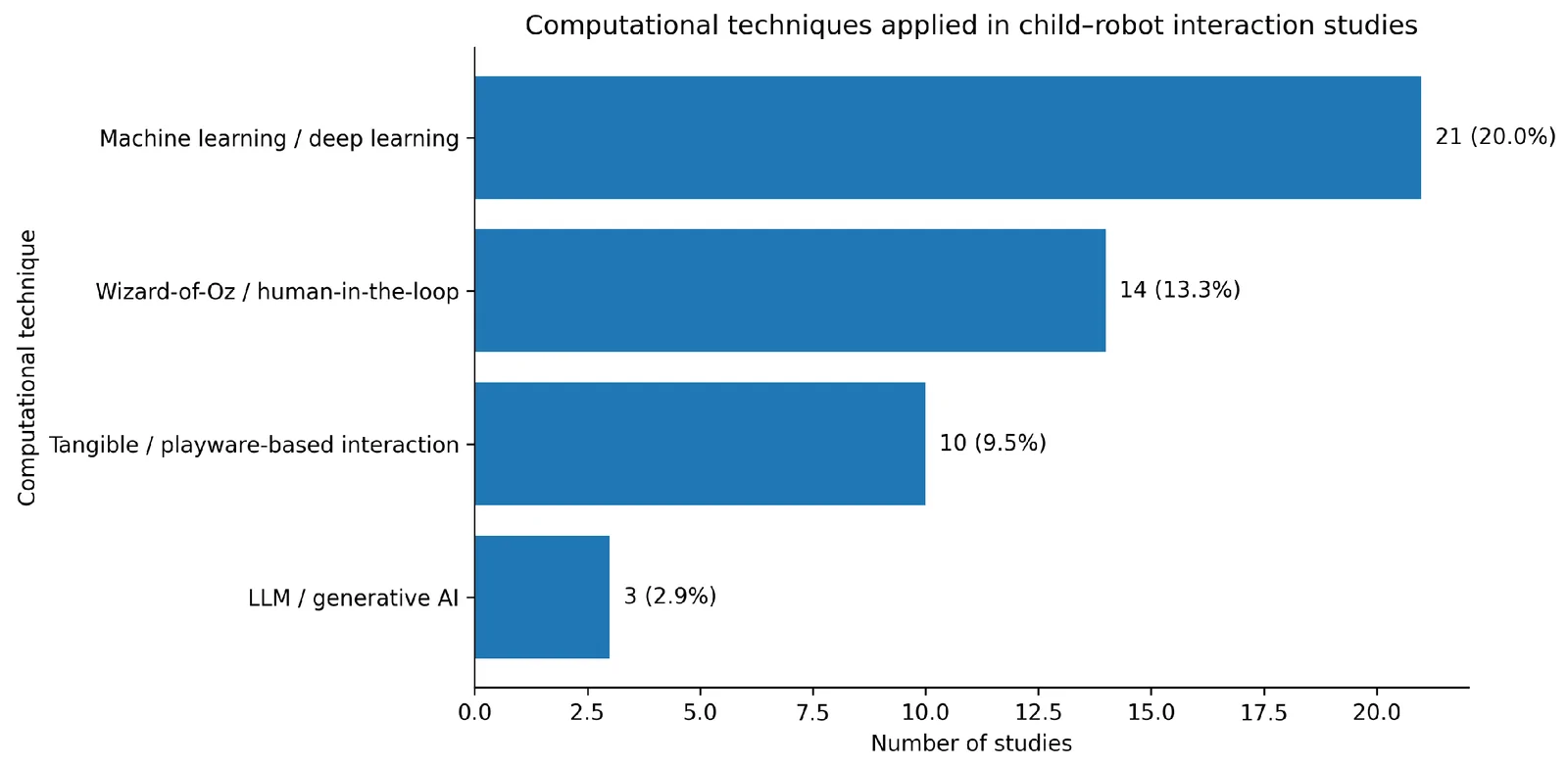
Computational techniques applied to support affective sensing, perception, and understanding in child–robot interaction.

Figure 7 presents the computational techniques applied to support affective sensing, perception, and understanding in child–robot interaction studies. The results show that machine learning and deep learning were the most frequently identified techniques, appearing in 21 studies (20.0%). These methods were mainly used for facial expression recognition, engagement detection, body movement analysis, stress recognition, and multimodal affect classification. Wizard-of-Oz or human-in-the-loop approaches were identified in 14 studies (13.3%), reflecting the continued use of supervised or semi-controlled interaction settings, particularly in early-stage prototypes and interventions with children. Tangible or playware-based computational interaction appeared in 10 studies (9.5%), while LLM/generative AI approaches were still emerging, appearing in only 3 studies (2.9%). Overall, the figure suggests that the field is still dominated by supervised learning, scripted interaction, and semi-autonomous approaches, whereas more adaptive computational techniques remain less represented.

To complement the analysis of computational techniques, the datasets supporting the training, validation, and evaluation of data-driven affective models were also examined. Table A1 summarizes the identified datasets in terms of population, role in the study, sample size, data modality, annotation or labeling strategy, availability, and main limitations. The analysis revealed substantial heterogeneity across studies, including the use of both public benchmark datasets and study-specific datasets. Importantly, some models subsequently applied in Child–Robot Interaction were trained or pre-trained using adult or general-population datasets, whereas child-specific datasets were generally smaller and often involved restricted age ranges or specific clinical populations. Dataset availability also varied, with some datasets publicly accessible and others restricted or with access conditions not reported. Annotation strategies ranged from expert or therapist coding to self-reported, automatically generated, and model-derived affect labels.

An important finding of the dataset-level analysis was the limited consistency of dataset reporting. While population, sensing modality, and affective labels could often be identified, information regarding dataset size, demographic composition, diversity, representativeness, and data-governance procedures was frequently incomplete or absent. Consequently, these characteristics could not be compared quantitatively across all studies. This reporting gap is particularly relevant because many of the reviewed articles focused primarily on robot interaction, intervention, or affect-recognition performance rather than on the construction and documentation of reusable datasets.

#### 4.2.5. RQ5: How Do Social Robots Express Emotions or Affective States During Interactions with Children?

Social robots expressed emotions or affective states mainly through embodied and multimodal behaviors. The most frequently reported expressive behaviors were gestures, body posture, and head or arm movements, identified in 82 studies (78.1%). These behaviors included waving, nodding, dancing, imitating movements, leaning, turning toward the child, or using posture to represent emotions such as happiness, sadness, anger, fear, or surprise. A second common channel was facial, LED, or screen-based expression, present in 65 studies (61.9%), especially in humanoid robots such as NAO, ZECA/Zeno, and Kaspar, which used facial expressions, eye LEDs, screen faces, or animated displays to communicate affective states [35,39,47]. Speech and dialogue were also widely used, appearing in 53 studies (50.5%), where robots expressed affect through verbal prompts, encouragement, feedback, storytelling, praise, questions, or emotionally framed dialogue [40,43].

In addition, robots expressed affect through movement, dance, navigation, or approach behaviors in 45 studies (42.9%) and through visual cues, images, tablets, or tangible interfaces in 32 studies (30.5%), particularly in emotion-recognition and storytelling tasks [39]. Less frequent expressive channels included sound, music, or sonification in 15 studies (14.3%) and haptic or tactile feedback in 11 studies (10.5%). Overall, the findings show that robot emotional expression in child–robot interaction is primarily multimodal, combining body movement, facial or visual displays, speech, and task-related feedback. The qualitative synthesis indicated that pre-programmed or scripted affective behaviors were common, whereas fully adaptive or autonomous emotional expression based on real-time interpretation of the child’s affective state was less consistently reported. Because autonomy level and real-time processing were not standardized quantitative variables in the extraction matrix, this observation should be interpreted qualitatively.

**Figure 8:**
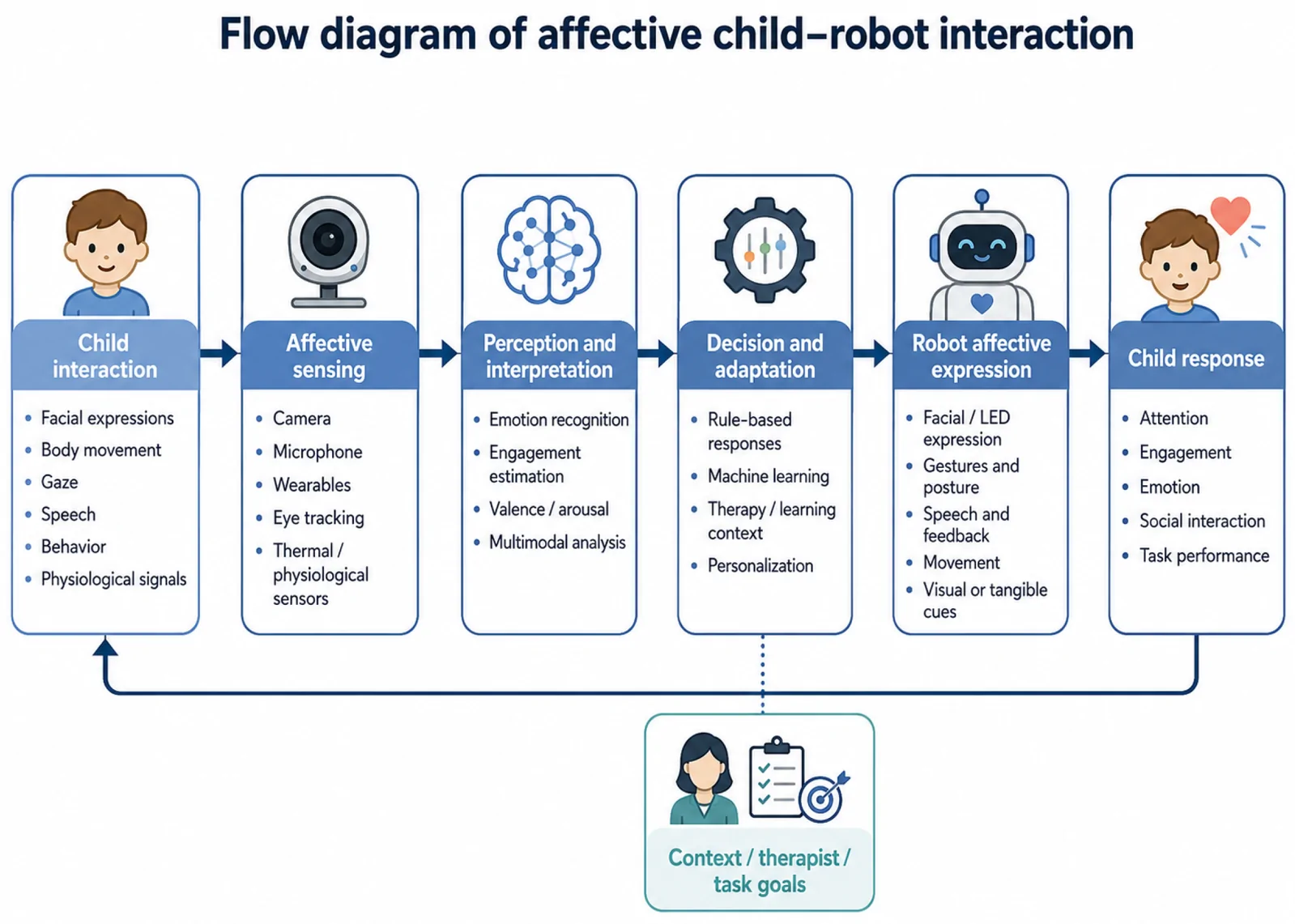
Flow diagram of affective child–robot interaction. The diagram summarizes the affective interaction loop from child signal capture to robot perception, adaptation, affective expression, and child response.

Figure 8 illustrates the general flow of affective child–robot interaction identified across the selected studies. The interaction begins with the child’s observable and physiological signals, including facial expressions, body movement, gaze, speech, behavior, and physiological responses. These signals are captured through affective sensing technologies such as cameras, microphones, eye tracking, wearable sensors, and thermal or physiological devices. The collected data are then processed through perception and interpretation mechanisms, including emotion recognition, engagement estimation, valence–arousal analysis, and multimodal analysis. Based on this interpretation, the robot adapts its behavior according to rule-based responses, machine learning models, therapeutic or educational goals, and personalization strategies. The robot then expresses affective states through facial or LED expressions, gestures, posture, speech, movement, and visual or tangible cues. Finally, the child’s response—expressed through attention, engagement, emotion, social interaction, or task performance—feeds back into the interaction loop, creating a continuous affective cycle. The diagram also highlights the role of the therapist, educator, or task context in guiding the robot’s decision-making and adaptation process.

#### 4.2.6. RQ6: How Are Engagement, Emotional Response, Acceptance, or Adaptive Behavior Evaluated in Affective Child–Robot Interaction Studies?

Engagement, emotional response, acceptance, and adaptive behavior were evaluated mainly through behavioral observation, affective signal analysis, task performance, questionnaires, qualitative reports, and computational classification. Engagement was one of the most frequent constructs, appearing in 46 studies (43.8%), and it was commonly assessed through children’s attention, gaze direction, gaze duration, participation, response to robot prompts, willingness to continue, and interaction quality. Emotional response was mainly evaluated through emotion recognition tasks, identified in 60 studies (57.1%), as well as through facial expressions, body movement, gestures, vocal responses, and physiological indicators. Social interaction was evaluated in 36 studies (34.3%), often through turn-taking, imitation, joint attention, social communication, and collaborative task behaviors.

The most common evaluation strategy was behavioral coding, either from direct observation or video recordings. Studies coded children’s gaze toward the robot or task object, duration of attention, number of correct/incorrect responses, response time, help requests, task completion, and whether the child wanted to continue the activity. For example, several robot-assisted therapy and playware studies evaluated engagement through gaze behavior, prompts, answers, and interaction persistence, as observed in studies using ZECA/Zeno, NAO, Kaspar, and tangible interfaces such as PlayBrick.

A second evaluation strategy involved computational analysis of affective signals. Machine learning or deep learning methods appeared in 21 studies (20.0%) and were used to classify engagement, emotion, affective state, or interaction quality from facial expressions, body posture, gaze, voice, or multimodal data. Physiological analysis was used in 15 studies (14.3%), especially when studies aimed to evaluate stress, arousal, attentiveness, or emotional regulation through EDA, BVP, heart rate, heart rate variability, thermal data, or wearable sensors. Examples include studies on physiological response and stress detection during robot-assisted autism interventions, such as [40,41,48].

Acceptance and perceived quality of interaction were evaluated less frequently than engagement and emotion recognition. These dimensions were usually measured through questionnaires, interviews, preference measures, willingness to interact again, perceived trust, comfort, or qualitative feedback from children, parents, teachers, or therapists. In some studies, acceptance was inferred indirectly through children’s sustained participation, positive affect, enjoyment, or preference for a robot condition.

Table 6 summarizes the main evaluation dimensions used in affective child–robot interaction (CRI) studies. Adaptive behavior was evaluated through how the robot adjusted or could potentially adjust its behavior according to the child’s affective or interaction state. This included personalized machine learning, affect-aware tutoring, rule-based adaptation, therapist-guided adaptation, and Wizard-of-Oz control. Wizard-of-Oz or human-in-the-loop approaches appeared in 14 studies (13.3%), showing that many systems still relied on partial human supervision to adapt robot behavior safely and flexibly. Overall, the evidence suggests that affective Child–Robot Interaction studies evaluate engagement and emotional response mainly through observable behavior and emotion-recognition outcomes, while acceptance and adaptive behavior are assessed through a combination of subjective reports, interaction performance, physiological data, and computational models [27].

#### 4.2.7. RQ7: What Methodological, Technical, Theoretical, and Ethical Gaps Remain in the Development of Affective Computing Approaches for Social Robotics with Children?

Across the selected studies, several gaps were identified from the coded corpus and the qualitative critical appraisal. At the level of affective representation, discrete emotion categories remained dominant, appearing in 60 studies (57.1%), whereas dimensional representations based on valence, arousal, or stress were identified in 21 studies (20.0%). At the computational level, machine learning and deep learning approaches were reported in 21 studies (20.0%), while Wizard-of-Oz or human-in-the-loop approaches appeared in 14 studies (13.3%), indicating that partial human control remained relevant in a subset of affective CRI systems. The sensing analysis also showed an imbalance toward externally observable information, with body-related features reported in 72 studies (68.6%) and facial features in 68 studies (64.8%), compared with physiological features in 18 studies (17.1%) and thermal features in 20 studies (19.0%). These corpus-level findings indicate an imbalance toward discrete affective representations, externally observable behavioral information, and relatively limited use of physiological and thermal sensing.

Beyond these frequency-based findings, the qualitative critical appraisal identified recurrent concerns related to small samples, short interaction periods, limited ecological validation, incomplete reporting of privacy and data-governance practices, and limited attention to explainability and long-term adaptation. Because sample size, interaction duration, real-time versus offline processing, and level of robot autonomy were not extracted as standardized quantitative variables across the 105 studies, these aspects are interpreted as qualitative observations rather than corpus-level prevalence estimates.

A further technical and ethical gap concerns the limited consideration of model interpretability and explainability [49]. As ML/DL-based affect recognition becomes increasingly connected to adaptive robot behavior, the absence of transparent decision mechanisms makes it difficult for therapists, educators, and caregivers to understand why a particular affective state was inferred or why a corresponding robot response was selected [50].

### 4.3. Taxonomy of Affective Computing in CRI

Building on the distinctions identified in previous reviews and using the hybrid deductive-inductive procedure described above, the 105 included studies were organized into six interconnected dimensions. The taxonomy consolidates several categories already present in the literature while extending them into an integrated representation of the affective interaction process in CRI.

The taxonomy was constructed after completion of data extraction using a hybrid deductive–inductive procedure. The unit of analysis was each of the 105 studies included in the final corpus. The deductive component was based on the research questions and the predefined extraction fields, which provided the initial analytical domains for organizing affective Child–Robot Interaction. The inductive component consisted of examining the coded values and recurrent patterns observed across the studies and grouping conceptually related categories within these domains.

The taxonomy development followed three stages. First, the standardized extraction matrix was reviewed to identify variables directly related to the affective interaction process, while descriptive bibliographic variables were excluded. Second, related codes were grouped according to their functional role in affective CRI, distinguishing interaction context, sensing modalities and observed features, affective constructs, computational and control mechanisms, robot expression, and evaluation/adaptation. Third, the resulting categories were reviewed against the complete corpus to refine boundaries, merge overlapping codes, and ensure coverage of the 105 included studies.

This process resulted in six core dimensions: (1) interaction context, (2) sensing modalities and observed features, (3) affective constructs and representation models, (4) computational and control mechanisms, (5) robot affective expression, and (6) evaluation and adaptation strategies. The taxonomy was not intended to assign each study to a single mutually exclusive class. Instead, studies could map to multiple subcategories within a dimension because affective CRI systems frequently combine several sensing modalities, computational approaches, expressive channels, or evaluation strategies. When information for a particular dimension was not reported, the study was coded as not reported rather than being assigned to a category by inference.

**Figure 9:**
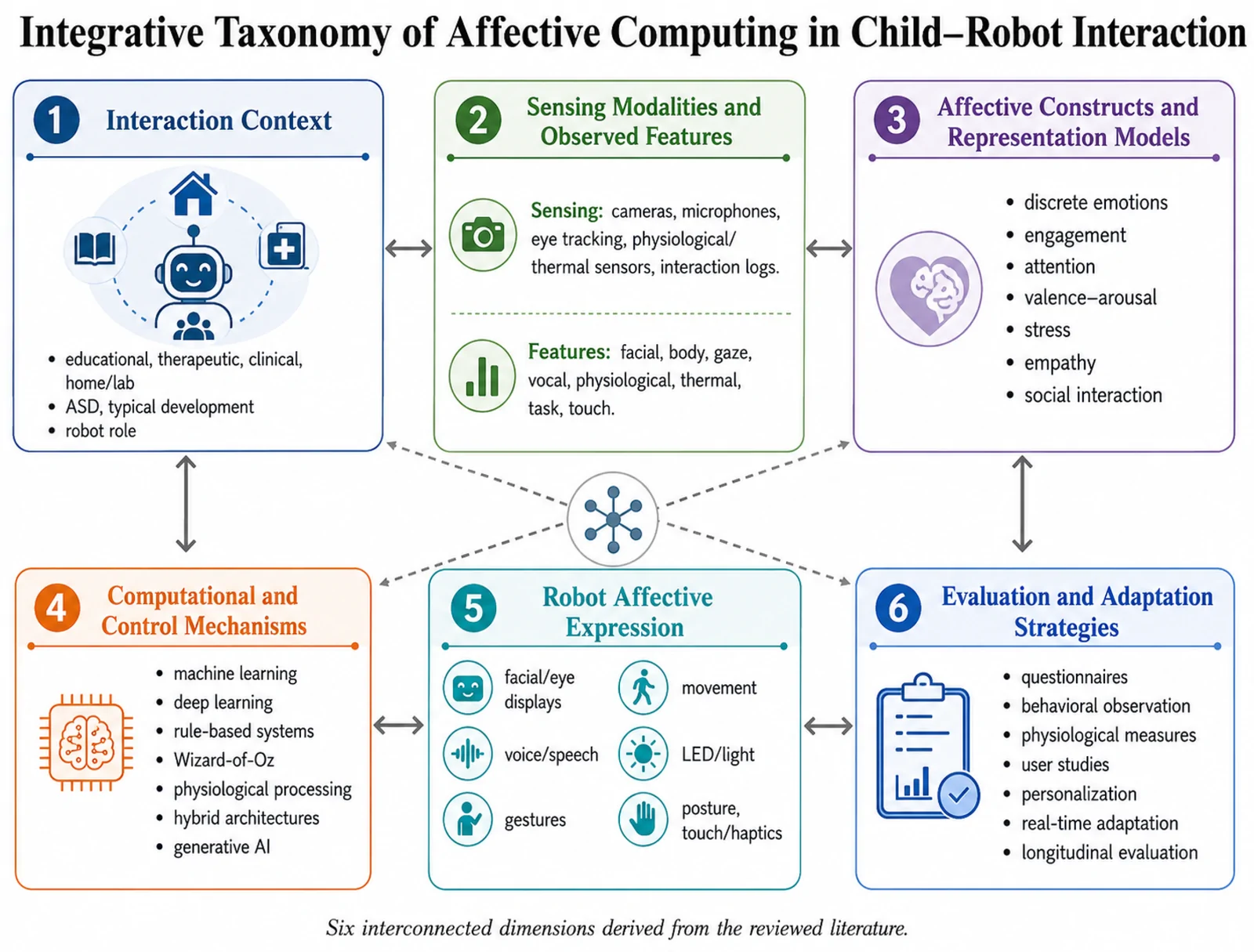
Taxonomy of Affective Computing in Child–Robot Interaction.

Figure 9 presents the taxonomy of affective computing in CRI, organized into six main dimensions. First, the interaction context defines whether the study is situated in autism intervention, education, therapy, social–emotional learning, or general child–robot interaction. Second, sensing modalities and observed features distinguish how affect-relevant information is acquired from the measurable behavioral, physiological, and interaction features derived from these sources. Sensing modalities include RGB/RGB-D cameras, microphones, eye-tracking systems, physiological sensors, thermal imaging, interaction logs, and tangible or touch sensors, whereas observed features include facial features, body movement, gaze behavior, vocal characteristics, physiological responses, thermal variation, task behavior, and touch interaction. Third, affective constructs and representation models describe how affect is conceptualized, including discrete emotions, engagement, attention, valence–arousal, stress, empathy, and social interaction. Fourth, computational and control mechanisms include machine learning, deep learning, rule-based systems, Wizard-of-Oz control, physiological signal processing, hybrid architectures, and emerging generative AI approaches. Fifth, robot affective expression refers to how robots communicate affect through gestures, facial or LED expressions, posture, speech, movement, visual cues, sound, or haptic feedback. Finally, evaluation and adaptation strategies describe how studies assess engagement, emotional response, acceptance, learning, social interaction, and the robot’s capacity to adapt to the child’s affective or interaction state.

To make the taxonomy construction process explicit and reproducible, Table 7 summarizes the operational definition and scope of each of the six taxonomy dimensions. These boundaries were established to distinguish the functional role of each category within affective Child–Robot Interaction and to reduce overlap during study mapping. The table also provides representative examples of the codes assigned to each dimension, clarifying how the extracted information from the 105 included studies was organized during the taxonomy refinement process.

## 5. Discussion and Limitations

### 5.1. Discussion

This review synthesized 105 selected studies on affective computing in child–robot interaction. The findings show that the field is strongly oriented toward autism-related contexts, with 72 studies (68.6%) focused on autism spectrum disorder (ASD). This confirms that social robots are frequently explored as therapeutic, educational, or social-support tools for children with neurodevelopmental needs. However, the results also show that affective computing in CRI is still predominantly used for emotion recognition, which appeared in 60 studies (57.1%), and for engagement-related analysis, identified in 46 studies (43.8%). Thus, much of the field remains centered on detecting or classifying affective states rather than fully understanding how affect should guide meaningful, adaptive, and developmentally appropriate robot behavior.

A central finding is the dominance of visually observable and behavioral features. Body movement, gesture, or posture appeared in 72 studies (68.6%), while facial expressions and facial features appeared in 68 studies (64.8%). Although these features are useful and technically accessible, their predominance raises important concerns, especially in ASD-related research [6,45,47]. Camera-based facial emotion recognition should not be treated as a sufficient or universally reliable method for monitoring affect in children with ASD. Neurodivergent children may display affect through heterogeneous combinations of gaze, body movement, vocal behavior, task engagement, and physiological responses, which may not correspond consistently to facial-expression patterns represented in conventional FER datasets. Recent reviews of emotion-recognition technologies for autism similarly indicate that facial-expression analysis remains predominant, while physiological and multimodal sensing are comparatively less explored despite their potential to capture complementary aspects of affective response. Thus, multimodal sensing may provide a more inclusive basis for affect estimation by reducing dependence on a single behavioral channel [41,48].

Beyond these established modalities, alternative sensing technologies may provide complementary information that is less dependent on overt facial expression. Infrared thermal imaging, for example, can capture peripheral temperature variations associated with autonomic activity. Recent evidence suggests that thermal responses from facial regions may provide information related to arousal, stress, and emotion regulation in children, including populations with neurodevelopmental conditions [7,51,52]. However, thermal imaging should not be interpreted as a direct or standalone measure of emotion, since thermal responses are influenced by environmental conditions, individual physiology, sensor placement, and experimental context. Its value may therefore lie primarily in combination with behavioral and physiological information rather than as an independent affect classifier [53,54].

A more robust direction is likely to involve multimodal affect assessment combining complementary behavioral and physiological information, including body posture and movement, gaze, speech and vocal prosody, task behavior, EDA, heart rate and HRV, BVP, thermal responses, and, where appropriate, spectral imaging. Such multimodal architectures may help compensate for ambiguity or failure in individual channels and provide a more inclusive representation of children’s affective states. At the same time, increasing the number of sensing modalities introduces additional challenges related to sensor synchronization, missing data, calibration, participant burden, privacy, data governance, and interpretability. Therefore, future affect-aware robots should not simply incorporate more sensors, but should determine which combinations provide reliable, developmentally appropriate, minimally intrusive, and explainable evidence for a given educational or therapeutic context.

The results also suggest that the role of the robot in CRI is not always clearly defined. Robots are often described as tutors, therapeutic assistants, social mediators, peers, companions, or engagement facilitators. The qualitative appraisal indicated that the interaction strategies associated with each robot role were not consistently specified. For example, if the robot acts as a tutor, its strategies should involve scaffolding, feedback, prompting, and learning progression. If it acts as a therapeutic assistant, strategies should emphasize predictability, therapist-guided adaptation, reinforcement, and gradual exposure to social cues. If it acts as a companion or peer, strategies should focus on reciprocity, turn-taking, emotional co-regulation, shared attention, and child agency. The lack of alignment between robot role and interaction strategy makes it difficult to determine whether the robot is supporting learning, therapy, social participation, emotional regulation, or merely functioning as an engaging technological interface [55,56].

This issue is particularly relevant for studies involving children without ASD. While autism-related studies tend to frame the robot as a therapeutic or intervention mediator, studies with neurotypical children often present robots as tutors, storytellers, companions, or social agents without fully explaining what socio-emotional role the robot should play. This is important because children are still developing emotional understanding, empathy, self-regulation, and social competence. Therefore, affective CRI should not only examine whether children recognize robot emotions or remain engaged, but also how robots can support emotional development in age-appropriate, ethical, and pedagogically meaningful ways.

The review also highlights a distinction between perception-centric and interaction-centric challenges in affective child–robot interaction. Perception-centric studies focus on recognizing or estimating affect from multimodal signals such as facial expressions, body movement, gaze, voice, behavior, and physiological data. These approaches are evident in studies using facial and body-based affect recognition, multimodal sensing, engagement estimation, physiological signals, gaze analysis, and personalized affect recognition models [11,33,34,40,43,57]. Interaction-centric studies, by contrast, examine how affective information can inform robot behavior adaptation, personalization, feedback, and long-term engagement strategies, as observed in adaptive emotional expression, affect-aware tutoring, Wizard-of-Oz control, personalized robot perception, and hybrid robot-interface interventions [39,43,58,59]. The qualitative synthesis suggests that research has advanced more clearly in affect perception than in the explicit specification of how affective information should guide real-time robot adaptation. This gap is also consistent with embodied emotion perspectives, which argue that emotions should be understood as situated, embodied, and action-oriented processes rather than as isolated perceptual labels [27].

A deeper ethical concern in affective Child–Robot Interaction involves the collection, storage, and long-term retention of children’s multimodal data. Affective CRI studies may capture facial video, voice, gaze, behavioral traces, thermal information, and physiological signals, often in combination. These data can remain sensitive long after the interaction itself because they may reveal not only identity-related information but also behavioral, developmental, emotional, or health-related characteristics. Therefore, ethical approval and parental consent should not be treated as sufficient safeguards on their own. Data governance should address the complete data lifecycle, including purpose specification, minimization of raw-data collection, retention periods, conditions for secondary use, access permissions, and secure deletion. Under the GDPR, personal data should be limited to what is necessary for the stated purpose and should not be retained in identifiable form longer than necessary, although longer retention for scientific research may be permitted when appropriate safeguards are implemented.

Facial emotion recognition and video-based affect analysis require particular caution in child populations. Identifiable facial video constitutes personal data, and its reuse may create risks that extend beyond the original research purpose, including secondary analysis, profiling, or re-identification. Importantly, facial images are not automatically classified as special-category biometric data under the GDPR; they become biometric data in the relevant legal sense when they are subject to specific technical processing that allows or confirms the unique identification of an individual. Nevertheless, facial video used in affective CRI remains highly sensitive, especially when combined with health-related, physiological, or developmental information. This distinction is important because the ethical risk of affective video does not depend only on whether it meets the legal definition of biometric identification, but also on how identifiable, linkable, and reusable the collected data remain.

The management of multimodal datasets also raises challenges for anonymization. In many affective CRI studies, complete anonymization may be difficult because facial appearance, voice, movement patterns, physiological traces, timestamps, and contextual information can jointly increase the possibility of re-identification. Pseudonymization can substantially reduce risk, but pseudonymized data remain personal data under the GDPR. Consequently, secure dataset management should combine pseudonymization with measures such as encryption, separation of identifying keys from research data, role-based access control, access logging, secure transfer, and clearly defined retention and deletion schedules. Where possible, researchers should also consider storing derived features rather than identifiable raw video or audio when the original signals are no longer necessary for the scientific objective.

From a regulatory perspective, affective CRI systems should therefore be designed according to data-protection-by-design and data-protection-by-default principles, with technical and organizational safeguards incorporated from the outset rather than added after data collection. The GDPR also requires a data protection impact assessment when processing involving new technologies is likely to create a high risk to individuals’ rights and freedoms, a condition that may be particularly relevant when identifiable multimodal data are collected from children. This concern is reinforced by the EU Artificial Intelligence Act, which prohibits the use of AI systems to infer emotions in educational institutions, except where such use is intended for medical or safety reasons. This distinction is highly relevant for affective CRI because educational, therapeutic, and clinical applications may be subject to different regulatory constraints even when they use similar sensing and inference technologies.

A related challenge concerns the interpretability and explainability of the computational models used in affective CRI. Machine learning and deep learning approaches can support increasingly sophisticated affect recognition and adaptive behavior; however, their predictions may be difficult for users to interpret when the underlying decision process remains opaque [60]. Previous HRI research has shown that exposing aspects of a robot’s internal decision process can improve users’ understanding of robot behavior, while self-explaining robot architectures have demonstrated the feasibility of generating explanations linked to the robot’s internal decision-making process [49,61]. This limitation becomes particularly relevant when affect recognition is subsequently used to modify the robot’s behavior.

Explainability is especially important in interactions involving children because explanations need to be developmentally appropriate and adapted to the user’s level of understanding. Previous work has investigated personalized robot-action explanations for children and adults [50], while more recent research in Child–Robot Interaction has explicitly explored multimodal explanation strategies and child-centered approaches to robot explainability [62,63]. Thus, an affect-aware robot should ideally communicate not only what affective state it inferred, but also, at an appropriate level of abstraction, what information supported the inference and why a particular adaptive response was selected.

However, explainability in Child–Robot Interaction also presents specific developmental challenges. Children are still developing the cognitive and socio-epistemic capacities needed to evaluate information reliability, recognize uncertainty, distinguish between system confidence and factual certainty, and understand the limitations of AI-based predictions [63]. Consequently, explanations designed for adults cannot be directly transferred to children. Explainable affective robots should therefore provide age-appropriate, simple, and context-sensitive explanations, while avoiding overconfidence, anthropomorphic framing, or representations that may lead children to overestimate the robot’s understanding or authority. In educational and therapeutic settings, this further supports the need for human oversight by parents, educators, or therapists, particularly when affective inferences are used to adapt robot behavior or influence intervention decisions.

An important ethical concern is how parents, caregivers, and educators may perceive the role of the robot in the child’s social life [64]. While robots are often presented as tutors, companions, therapeutic assistants, or social mediators, it is not always clear whether they are designed to enhance human social interaction or whether they risk becoming a substitute for it. This concern is especially relevant when robots are highly predictable, responsive, and emotionally expressive, because some children may prefer interacting with the robot rather than engaging with peers, teachers, therapists, or family members. In this sense, the ethical question is not only whether the robot is accepted by the child but whether it contributes to broader social participation and emotional development [12,65].

The intention to design robots for long-term emotional interaction with children, and even assign them roles such as tutors, caregivers, companions, or friends, raises important questions about the quality and boundaries of the emotional communication these robots can provide [12,66]. It is well established that humans tend to respond socially and emotionally to information and communication technologies, even when they are aware that they are interacting with machines [67]. In the case of children, this issue becomes even more relevant because they are still developing empathy, emotional understanding, self-regulation, and social relationships. Therefore, social robots should not be evaluated only in terms of engagement, acceptance, or task performance, but also in terms of how their affective behavior influences children’s emotional development and social participation. If robots are designed as tutors or therapeutic mediators, their emotional expressions and responses should support learning and human interaction; however, if they are framed as friends or substitute companions, ethical concerns arise regarding attachment, dependency, and possible social isolation. This suggests that affective CRI should clearly define the robot’s role and ensure that long-term emotional interaction with robots functions as a bridge toward human relationships rather than replacing them.

The findings can also be interpreted through developmental and cognitive theories that help explain why affective CRI should move beyond automatic emotion recognition. From a sociocultural perspective, the robot may act as a mediator or scaffolding agent that supports the child’s learning within guided interaction, rather than as an isolated technological tool [68]. Social learning theory is also relevant because children may learn emotional expressions, social cues, and regulatory strategies by observing and interacting with the robot; therefore, the robot’s emotional expressiveness must be developmentally appropriate and not cognitively overwhelming [69]. In addition, theories of emotion regulation and co-regulation suggest that the robot should not only classify affective states, but also support the child in understanding, expressing, and regulating emotions [70,71]. This is particularly important in ASD contexts, where affective signals may not follow neurotypical patterns and where visual facial-emotion recognition alone may be insufficient. Embodied cognition further supports the need for multimodal approaches, since affect is expressed through the body, gaze, movement, behavior, physiology, and context, not only through facial expressions [27,72,73]. Finally, ecological and social-presence perspectives highlight the ethical need to situate the robot within a broader network of parents, teachers, therapists, and peers, ensuring that the robot functions as a bridge to human interaction rather than as a substitute for social relationships [70,74].

Another relevant design concern emerging from the reviewed studies is the limited clarity regarding the appearance and emotional expressiveness of social robots for children [6,55]. Although humanoid robots were the most frequently used morphology in the selected corpus, robot design for children should not necessarily pursue a highly human-like appearance. The uncanny valley hypothesis suggests that robots that appear almost human, but not fully natural, may generate discomfort or ambiguity rather than trust or affinity [75]. This is particularly important in child–robot interaction, where children are still developing emotional understanding, empathy, and social interpretation [56]. Robots can express affect through different strategies [76], including animated facial expressions on screens, mechanical movements of facial features such as eyes, mouth, eyebrows, or eyelids, LEDs, body posture, gestures, movement, or additional features such as ears in animal-like platforms [77]. However, the literature does not clearly establish which expressive components are most relevant, interpretable, or developmentally appropriate for children. Some robots use symbolic affective cues, such as LED matrices, heart icons, or color changes to represent emotional states, but these mappings may not be self-evident; for example, associating sadness with blue or anger with red may depend on cultural, developmental, or contextual interpretation rather than being universally understood. Therefore, robot emotional expression should not only be technically feasible, but also emotionally meaningful, culturally interpretable, and cognitively manageable for children. From an embodied emotion perspective, robot affect should be understood as part of situated interaction involving perception, action, body, context, and communication, rather than as an isolated visual display. Future studies should therefore evaluate how robot appearance, morphology, expressive modality, timing, intensity, and control algorithms influence children’s interpretation of robot emotions, while avoiding overly human-like or ambiguous designs that may increase discomfort, confusion, or emotional overload [27].

Therefore, future affective CRI research should address two connected questions: what signals should the robot capture from the child, and how should the robot communicate its own affective state back to the child? Emotional expression is a central part of child–robot communication, but it is not the whole interaction. A robot’s affective behavior must be coordinated with its role, the task goal, the child’s developmental stage, and the interaction context [78]. For instance, a tutor robot may require expressive feedback that supports learning and attention, whereas a therapeutic robot may require predictable, low-intensity, and carefully timed affective cues that avoid emotional overload. Current studies often report that robots use gestures, facial expressions, LEDs, speech, or movement, but they provide limited explanation of how these modalities are selected, sequenced, controlled, or validated with children. This suggests that future work should evaluate not only emotion recognition accuracy but also the interpretability, intensity, timing, and appropriateness of robot emotional expressions as part of a broader affective communication process.

Geographical and cultural diversity represents an additional limitation of the available evidence. Research production was concentrated in a relatively small number of countries, with the United States accounting for 20.0% of the corpus and Portugal and Italy for 9.5% each. This concentration is particularly relevant in affective CRI because emotional expression, gaze behavior, interpersonal distance, anthropomorphism, interpretation of robot gestures, and expectations regarding child–adult or child–technology interaction may vary across cultural contexts. Consequently, affective models, robot behaviors, and evaluation instruments validated within a limited cultural setting should not be assumed to generalize directly to children from other linguistic or cultural backgrounds. Future research should therefore prioritize cross-cultural validation, culturally adapted affective measures, multilingual interaction, and more geographically diverse child populations.

### 5.2. Limitations

This review has several limitations. First, the analysis was based on a selected corpus of 105 included studies, which provides a focused synthesis of affective computing in child–robot interaction, but it does not represent the entire literature on social robotics, HRI, or affective computing. In addition, the included studies were heterogeneous in terms of populations, robot platforms, interaction contexts, methodological designs, affective signals, computational techniques, and evaluation measures, which limits direct comparison across studies.

Second, the corpus was strongly oriented toward ASD-related studies, which may bias the interpretation of affective CRI toward therapeutic and intervention-based contexts. Although this focus is relevant, less evidence is available on how social robots should support emotional development, empathy, self-regulation, and social interaction in children without ASD.

Third, another limitation of the reviewed evidence concerns the datasets supporting data-driven affective models. A substantial proportion of the identified datasets were study-specific collections, while access conditions were heterogeneous: some datasets were publicly available, some were restricted, and for several datasets availability was not reported. Publicly available child-specific datasets were comparatively scarce, limiting reproducibility, external validation, and direct comparison across computational approaches. Furthermore, the datasets were predominantly centered on observable visual cues, particularly facial expressions and body posture or movement, whereas datasets incorporating physiological signals were less common. Although some studies collected EDA, heart rate, HRV, BVP, skin temperature, or related physiological measures, these datasets generally involved small samples and were frequently study-specific or unavailable for reuse. This imbalance may constrain the development of multimodal models capable of capturing internal affective states such as arousal, stress, and emotional regulation. This limitation is further compounded by the use, in some studies, of adult or general-population datasets to train or pre-train models subsequently applied to children, raising concerns regarding developmental representativeness and generalizability.

A related limitation concerns the inconsistent reporting of privacy and data-governance practices across the reviewed studies. Although ethics approval and parental consent were commonly reported, detailed information on retention periods, anonymization or pseudonymization, encryption, access control, secondary use, deletion, and cross-institutional data sharing was often absent. Consequently, the review could not systematically assess compliance with data-protection-by-design principles or determine whether multimodal child datasets were managed according to comparable privacy standards.

Finally, the role and emotional expressiveness of the robot were not always clearly defined. Many studies describe robots as tutors, companions, therapeutic assistants, or social mediators, but do not specify which interaction strategies or control algorithms are appropriate for each role. This raises both methodological and ethical concerns, especially in long-term interactions where robots should support, rather than replace, human relationships. Future research should therefore clarify the robot’s role, evaluate its emotional expressions, and ensure that affective CRI promotes children’s social and emotional development in safe and ethically grounded ways.

## 6. Conclusions

This review synthesized 105 selected studies on affective computing in child–robot interaction (CRI), showing that the field has grown significantly in recent years and is strongly oriented toward autism-related contexts. Most studies focused on children with ASD, emotion recognition, engagement, social interaction, and therapeutic or educational support. The results indicate that social robots are increasingly used as tutors, therapeutic assistants, social mediators, companions, and affective interaction partners, but their role is not always clearly defined in relation to the interaction strategies used.

Affective computing approaches in CRI remain largely perception-centric, with a strong emphasis on facial expression recognition, body movement, gaze, and behavioral indicators. Although these modalities are useful, relying mainly on camera-based emotion recognition is limited, particularly for children with ASD, whose emotional expressions may differ from neurotypical patterns. Therefore, future systems should move toward multimodal affective sensing, integrating facial expressions, body posture, gaze, voice, physiological signals, task behavior, contextual information, and observations from therapists, teachers, or caregivers.

The review also shows that computational techniques are still developing. Machine learning and deep learning methods were identified in 21 studies (20.0%), while Wizard-of-Oz or human-in-the-loop approaches appeared in 14 studies (13.3%). The qualitative appraisal additionally suggested recurrent use of scripted or semi-autonomous interaction and short-duration protocols, although these characteristics were not systematically quantified across the complete corpus.

Another key conclusion is that affective CRI should not be reduced to automatic emotion recognition. Children are still developing emotional understanding, empathy, self-regulation, and social competence; therefore, robot emotional expressiveness must be developmentally appropriate, interpretable, and ethically safe. Robots should support children’s emotional learning and social participation without replacing human relationships. This is especially relevant in long-term interactions, where parents, teachers, and therapists may be concerned about attachment, dependency, privacy, or possible social isolation.

Overall, this review proposes a broader understanding of affective computing in CRI as a dynamic, embodied, relational, and developmental process. The taxonomy derived from the included studies highlights six interconnected dimensions: interaction context; sensing modalities and observed features; affective constructs and representation models; computational and control mechanisms; robot affective expression; and evaluation and adaptation strategies.

## Figures and Tables

**Table 1:** Positioning of the proposed taxonomy in relation to closely related reviews and frameworks.

Previous Work	Primary Scope	Relation to the Present Taxonomy	Contribution of the Present Review
[2]	Affective computing in Human–Robot Interaction	Provides a broad conceptual basis for affect recognition, context, and adaptive HRI.	Specializes these concerns to CRI and integrates them with child-specific sensing, affective representation, robot expression, and evaluation/adaptation.
[20]	Affective communication in socially assistive robots for children with ASD	Overlaps with affective representation, computational approaches, sensing, and robot emotional communication.	Extends the scope beyond ASD-specific affective communication and organizes the complete affective interaction process into explicit functional dimensions.
[7]	Affective computing techniques for infant-robot interaction	Strongly related to sensing and affect-recognition techniques.	Separates sensing modalities from observed features and connects perception with affective constructs, computational/control mechanisms, robot expression, and evaluation/adaptation.
[15]	Child engagement in digital and robotic activities	Provides a detailed perspective on engagement and its assessment.	Places engagement within a broader affective CRI framework that also includes sensing, affective representation, computational processing, robot expression, and adaptation.

**Table 2:** Boolean search strategy based on conceptual blocks.

Block	Concept	Search Terms
A	Affective computing	“Affective computing” OR “affective interaction” OR “emotion recognition” OR “emotion detection” OR “emotion expression” OR “emotional expression”
B	Robotics and interaction	“Child-robot interaction” OR “human-robot interaction” OR “social robot*” OR “educational robot*” OR “socially assistive robot*”
C	Child population	child* OR children OR ASD OR autism OR “autism spectrum disorder”

**Table 3:** Operational coding framework for the stages of affective processing in Child–Robot Interaction.

Information Family	Sensing Modality/ Data Source	Observed Feature or Behavior	Typical Inferred Construct
Facial	RGB/RGB-D cameras, video recordings	Facial expressions, facial landmarks, facial action units	Emotion, valence, engagement
Body	RGB/RGB-D cameras, video, behavioral observation	Body movement, posture, gesture, head movement	Emotion, engagement
Gaze	Eye-tracking devices or camera-based gaze estimation	Gaze direction, fixation duration, eye contact	Attention, engagement, joint attention
Vocal	Microphones and audio recordings	Speech, prosody, pitch, intensity, vocal characteristics	Emotion, engagement
Physiological	Wearable or contact-based physiological sensors	EDA, BVP, HR/HRV, ECG, EEG, skin temperature	Arousal, stress, attention, regulation
Thermal	Thermal or infrared imaging	Facial or peripheral temperature and temperature variation	Arousal, stress
Task/interaction	Game, task, or interaction logs	Accuracy, response time, prompts, help requests, persistence, completion	Engagement, learning
Touch/tangible	Touch sensors and tangible interfaces	Touch events, contact patterns, tangible interaction responses	Engagement, interaction

**Table 4:** Observed affect-relevant features and behaviors identified in the included studies.

Observed Affect-Relevant Feature/Behavior	*n*	%
Body movement/gesture/posture	72	68.6%
Facial expressions/facial features	68	64.8%
Task/interaction behavior	37	35.2%
Gaze behavior	24	22.9%
Vocal/speech-related features	23	21.9%
Thermal features	20	19.0%
Physiological features	18	17.1%
Touch/tangible interaction behavior	9	8.6%

**Table 5:** Affective constructs and representation models.

Affective Representation	*n*	%
Discrete emotions	60	57.1%
Engagement/attention	47	44.8%
Social interaction/empathy-related constructs	45	42.9%
Valence/arousal/stress	21	20.0%
PAD model	0	0%

**Table 6:** Evaluation dimensions in Child–Robot Interaction.

Evaluation Dimension	Main Indicators Used	Frequency in Selected Corpus
Emotion recognition/emotional response	Correct emotion labels, facial expression recognition, emotion imitation, affective classification	60 studies (57.1%)
Engagement	Attention, gaze, participation, response to prompts, task persistence, interaction quality	46 studies (43.8%)
Social interaction	Turn-taking, imitation, social communication, responsiveness, peer/robot interaction	36 studies (34.3%)
Learning/socio-cognitive outcomes	Emotional learning, theory of mind, social emotional learning, task improvement	45 studies (42.9%)
Stress/arousal	Physiological response, EDA, BVP, HR/HRV, thermal or wearable data	17 studies (16.2%)
Empathy/acceptance-related constructs, Join attention	Empathy, trust, perceived usefulness, willingness to interact, robot preference Gaze following, shared attention, response to join-attention cues	5 studies (4.8%)

**Table 7:** Operational definitions and boundaries of the taxonomy dimensions.

Taxonomy Dimension	Operational Boundary	Representative Examples
**Interaction context**	Defines the purpose, population, and setting in which affective child–robot interaction takes place.	ASD intervention, therapy, education, social–emotional learning, general child–robot interaction.
**Sensing modalities and observed features**	Distinguishes how affect-relevant information is acquired from the observable behavioral, physiological, or interaction features derived from these sources.	Sensing modalities/data sources: RGB/RGB-D cameras, microphones, eye-tracking systems, physiological sensors, thermal imaging, interaction logs, and tangible/touch sensors. Observed features/behaviors: facial features, body movement, gaze behavior, vocal features, physiological responses, thermal variation, task behavior, and touch interaction
**Affective constructs and representation models**	Describes how the child’s affective, emotional, or social state is conceptualized, represented, or categorized.	Discrete emotions, engagement, attention, valence–arousal, stress/arousal, empathy, social interaction.
**Computational and control mechanisms**	Includes methods used to process, interpret, classify, or integrate affective information and mechanisms used to control or adapt the interaction.	Machine learning, deep learning, multimodal fusion, rule-based systems, physiological signal processing, Wizard-of-Oz, human-in-the-loop approaches.
**Robot affective expression**	Refers to the output channels through which the robot communicates affective or emotional information to the child.	Gestures, body posture, facial or screen expressions, LEDs, speech, movement, sound, haptic feedback.
**Evaluation and adaptation strategies**	Describes how affective interaction outcomes are assessed and how the robot modifies its behavior according to the child’s affective or interaction state.	Engagement assessment, emotion recognition, learning outcomes, acceptance, personalization, adaptive feedback, behavioral adjustment.

## Data Availability

No new data were created or analyzed in this study. Data sharing is not applicable to this article.

## Supplementary Materials

The following supporting information can be downloaded at: https://www.mdpi.com/article/10.3390/s26185721/s1.

## Abbreviations

The following abbreviations are used in this manuscript:

HR/HRV	Heart Rate/Heart Rate Variability
EDA	Electrodermal Activity
BVP	Blood Volume Pulse
EEG	Electroencephalography
ECG	Electrocardiography
CNN	Convolutional Neural Network
PAD	Pleasure–Arousal–Dominance
FACS	Facial Action Coding System

## Appendix A

### Appendix A.1

**Table A1:** Characteristics of datasets used in selected studies.

Study	Dataset/Data Source	Population	Data Modality	Availability	Annotations/Labels
[32]	ISR-FABEL	Children	Facial + body landmarks; 2D/3D; RGB-D	Public release announced via repository	Psychology students; majority voting; 7 emotion classes
[79]	FER2013	Adults	Facial images, grayscale 48 × 48	Public	7 emotions
[79]	CAFE	Children	Facial images	Public	7 emotions
[33]	BabyRobot Emotion Database (BRED)	Children, 6–12 years	RGB video; facial expressions + 2D body posture	Public	6 emotions; hierarchical face/body/whole-body labels; multi-annotator majority vote
[33]	GEMEP	Adults	Face + body	Public	17 emotions
[33]	AffectNet	Adults	Face	Public	Neutral, Happiness, Anger, Sadness, Disgust, Contempt, Fear, Surprise, plus None, Uncertain and Non-face
[44]	Study-specific image dataset (unnamed)	Children with autism	Facial RGB images	Not reported	Oxford Emotion API scores for 8 emotions: anger, contempt, disgust, fear, happiness, neutral, sadness, surprise
[80]	CK+	General population; not child-specific	Facial images	Public	6 emotions: anger, disgust, fear, happiness, sadness, surprise; +neutral in 7-class configuration
[46]	Study-specific physiological dataset (unnamed)	Adolescents/Adults	EDA, BVP, skin temperature	NR	Experimental-condition labels: baseline, walking, traditional navigation, social navigation
[41]	Study-specific physiological dataset (unnamed)	Children with ASD, 11 years	EDA, BVP, HR, skin temperature	NR	Attentive/Inattentive
[36]	MDCA – Multimodal Dataset of Children with Autism	Children with ASD	Face, body, gaze, audio, HR, EDA, temperature, accelerometer	Available on request/restricted	Continuous valence, arousal, engagement [−1, +1]; 5 expert coders; gold standard from 3 highest-agreement coders
[78]	Study-specific contextual interaction dataset	Children, 8–10 y., Portugal	Game + social-context logs; questionnaire ratings	NR	Social engagement, help, self-validation, perceived affective interdependence; self-reported Likert ratings converted to binary classes
[78]	Inter-ACT corpus	Children	Contextualized child affective expressions; visual + contextual information	NR	Valence: positive/neutral/negative
[81]	Study-specific child video dataset (unnamed)	Arabic-speaking children with ASD, 5–10 years.	Facial video/68 facial landmarks	NR	Engaged/Not engaged;
[82]	Study-specific EDA emotion dataset (unnamed)	Children: 9 ASD + 7 TD	EDA, 32 Hz	Restricted/not shareable; requests to authors	Video-aligned manual event annotation; dominant states Calm, Frustrated, Happy, plus Unclear; activity/event labels S1/S2/S3
[83]	KDEF+Real Emotion	Adults	Face/RGB	NR	7 categorical emotions
[83]	EmoUERJ + ESD	Adults	Audio	Public	Multiple emotion categories; model uses angry, happy, surprise, neutral
[34]	DE-ENIGMA	children with ASD	RGB + depth; 3D skeleton/body pose	Public	37 child action classes; continuous valence and arousal annotated by 5 specialized therapists
[43]	Study-specific long-term multimodal ASD dataset (unnamed)	Children with ASD	Face/body, audio, game-performance	NR	Binary arousal and valence
[84]	Study-specific child facial-affect dataset (unnamed)	general-population children, 5–7 years.	Facial video (30 fps) + interaction/game data	NR	Automatic Affdex scores: 7 emotions, 21 facial expressions, engagement and valence; continuous scores rather than human labels

### Appendix A.2

**Table A2:** Included studies and coded characteristics of the final corpus ordered by publication year (*n* = 105).

Year	Citation	Title	Article Type	Population	Robot Platform/Morphology	Affect-Relevant Sensing/Features	Constructs/Methods
2026	[85]	Curiosity and Affect-Driven Cognitive Architecture for HRI	Journal article	No empirical participants/planned	NR/Humanoid	Face/FER; Gaze/eye; Tangible/touch; Thermal/IR	Constructs: Engagement; Social interaction; Learning. Methods: NR.
2026	[82]	Effects of a human-robot collaborative teaching approach on preschoolers’ social-emotional competence and learning behaviors	Journal article	Children general/non-ASD	Pepper/Humanoid	Body/gesture	Constructs: Social interaction; Learning. Methods: NR
2026	[86]	Guidelines for Emotion Recognition in Robot-Supported Interventions in Autism	Journal article	Children with ASD	Kaspar/Humanoid	Face/FER; Body/gesture; Voice/audio; Physiology; Gaze/eye	Constructs: Emotion recognition; Engagement; Stress/arousal; Valence. Methods: Physiological analysis; Review.
2026	[87]	Dot to Dot. Storytelling Without Words, Through Gesture and Abstraction: What Can Robotics Learn from Puppetry’s Language of Gesture? Insights from a Theatrical Experiment	Conference Paper	NR	NR	NR	Constructs: NR. Methods: NR.
2025	[32]	ISR-FABEL: A Landmark-Based Dataset and Multimodal Emotion Recognition Framework for Child-Robot Interaction	Journal article	Children general/non-ASD	NAO/Humanoid	Face/FER; Body/gesture; Task/game performance; Text/LLM	Constructs: Emotion recognition; Engagement; Learning. Methods: ML/DL; Statistical.
2025	[88]	Children’s Perception and Engagement with a Humanoid Robot Speaking Their Native Language	Conference paper	Children general/non-ASD	NR/Humanoid	Face/FER; Body/gesture; Voice/audio; Text/LLM; Thermal/IR	Constructs: Engagement; Social interaction; Learning. Methods: LLM/GenAI; Qualitative.
2025	[37]	A Child-Aware Setup for Automatic Evaluation of Interaction with Robots in NAO Autism Therapy	Conference paper	Children with ASD	NAO/Humanoid	Face/FER; Body/gesture; Gaze/eye; Text/LLM	Constructs: Engagement; Joint attention. Methods: ML/DL; Qualitative; Wizard-of-Oz.
2025	[89]	Gestures vs. Faces: Exploring Emotion Recognition in Child-Robot Interaction	Conference paper	Children general/non-ASD	NAO/Humanoid	Face/FER; Body/gesture; Thermal/IR	Constructs: Emotion recognition; Learning. Methods: Statistical.
2025	[90]	Evaluating the Correlation Between Social Robot NAO and Preschoolers’ Socio-Emotional and Cognitive Skills	Conference paper	Children general/non-ASD	NAO/Humanoid	Face/FER; Body/gesture; Physiology	Constructs: Emotion recognition; Empathy; Social interaction; Learning. Methods: Statistical; Physiological analysis.
2025	[76]	Can Robots Teach Emotions? Evaluating NAO’s Educational Role in Social and Emotional Learning	Conference Paper	Children general/non-ASD	NAO/Humanoid	Face/FER; Body/gesture; Physiology	Constructs: Emotion recognition; Engagement; Empathy; Learning. Methods: Statistical; Physiological analysis.
2025	[91]	Cognitive Socially Assistive Robot for Multimodal Assessment and Adaptive Support in Autism Therapy	Conference Paper	ASD population	NR	Face/FER; Body/gesture; Voice/audio; Gaze/eye; Task/game performance	Constructs: Emotion recognition; Engagement; Social interaction; Learning; Joint attention. Methods: ML/DL.
2025	[92]	The Impact of Robot-Assisted Picturebooks Reading on Kindergarteners’ Social Emotional Learning	Conference Paper	Children general/non-ASD	NR	Face/FER; Body/gesture	Constructs: Learning. Methods: NR.
2025	[93]	Emotionally Expressive Robots: Implications for Children’s Behavior toward Robot	Conference paper	Children general/non-ASD	NR	Face/FER; Body/gesture; Task/game performance; Thermal/IR	Constructs: Emotion recognition; Empathy; Social interaction. Methods: Wizard-of-Oz.
2025	[94]	Supporting Preschool Emotional Development with AI-Powered Robot	Conference paper	Children general/non-ASD	NR	Face/FER; Body/gesture; Voice/audio; Tangible/touch; Text/LLM	Constructs: Emotion recognition; Engagement; Learning. Methods: NR.
2025	[83]	Advancing Emotionally Aware Child-Robot Interaction with Biophysical Data and Insight-Driven Affective Computing	Journal article	Children general/non-ASD	NAO/Humanoid	Face/FER; Body/gesture; Voice/audio; Task/game performance; Text/LLM	Constructs: Emotion recognition; Engagement. Methods: ML/DL; Statistical.
2025	[95]	Designing socially assistive robots for clinical practice: insights from an asynchronous remote community of speech-language pathologists	Journal article	Children general/non-ASD	NAO/Humanoid	Face/FER; Body/gesture; Voice/audio	Constructs: Engagement; Social interaction. Methods: Qualitative; Wizard-of-Oz.
2025	[96]	Sally and… Cozmo? Training theory of mind using a toy robot: a pilot study	Journal article	Children with ASD	NR/Humanoid	Body/gesture; Task/game performance	Constructs: Emotion recognition; Engagement; Social interaction; Learning. Methods: Wizard-of-Oz.
2025	[97]	Design of JARI: A Robot to Enhance Social Interaction in Children with Autism Spectrum Disorder	Journal article	Children with ASD	NR/Animal-like/zoomorphic	Face/FER; Body/gesture; Gaze/eye; Task/game performance	Constructs: Emotion recognition; Social interaction. Methods: Qualitative; Wizard-of-Oz.
2025	[48]	Physiological Response in Children with Autism Spectrum Disorder (ASD) During Social Robot Interaction	Journal article	Children with ASD	Pepper/Humanoid	Face/FER; Body/gesture; Physiology	Constructs: Stress/arousal. Methods: Physiological analysis.
2024	[98]	Exploring Emotional and Cognitive Engagement in School Age Children: An In-Depth Analysis of Interaction with the NAO Social Robot During the Storytelling Activity	Conference	Children general/non-ASD	NAO/Humanoid	NR	Constructs: Engagement. Methods: NR.
2024	[42]	Heart Rate Predictive Value Using Wearable Sensors in Social Robotics Conversations to Help Children with Autism	Conference paper	Children with ASD	NAO/Humanoid	Face/FER; Physiology; Task/game performance; Tangible/touch	Constructs: Emotion recognition; Stress/arousal; Social interaction; Learning. Methods: ML/DL; Physiological analysis.
2024	[99]	TotCareBot: A Comprehensive Approach Towards Enhancing Early Childhood Education through Robotics	Conference Paper	Children general/non-ASD	NR	Body/gesture; Task/game performance; Thermal/IR	Constructs: Emotion recognition; Learning. Methods: NR.
2024	[100]	A HeARTfelt Robot: Social Robot-Driven Deep Emotional Art Reflection with Children	Conference paper	Children general/non-ASD	NR	Body/gesture; Voice/audio	Constructs: Emotion recognition; Engagement; Empathy; Learning. Methods: Statistical; Qualitative.
2024	[101]	How Preference Towards Robotic Agents Affects Choice Accuracy in Children with Autism Spectrum Disorder	Conference paper	Children with ASD	NR	Face/FER; Task/game performance; Tangible/touch; Thermal/IR	Constructs: Engagement. Methods: Qualitative.
2024	[102]	Sustained attention in children with ASD: A comparative analysis between traditional intervention and the use of the MyRoT robotic tree	Conference Paper	Children with ASD	NR	Face/FER; Body/gesture; Task/game performance	Constructs: Emotion recognition; Social interaction. Methods: ML/DL; Statistical; Wizard-of-Oz.
2024	[59]	An Empathetic Social Robot with Modular Anxiety Interventions for Autistic Adolescents	Conference paper	Children with ASD	Pepper/Humanoid	Face/FER; Body/gesture; Voice/audio; Task/game performance; Text/LLM; Thermal/IR	Constructs: Emotion recognition; Engagement; Stress/arousal; Empathy. Methods: LLM/GenAI; Statistical.
2024	[103]	Human-Robot Mutual Learning Through Affective-Linguistic Interaction and Differential Outcomes Training	Conference paper	Children general/non-ASD	NR	Face/FER; Voice/audio; Tangible/touch; Text/LLM	Constructs: Social interaction; Learning. Methods: LLM/GenAI; Statistical; Qualitative.
2024	[57]	Challenges in Observing the Emotions of Children with Autism Interacting with a Social Robot	Journal article	Children with ASD	Kaspar/Humanoid	Face/FER; Body/gesture; Voice/audio; Physiology; Gaze/eye	Constructs: Emotion recognition; Engagement; Stress/arousal. Methods: Physiological analysis.
2024	[104]	The use of social robots as teaching assistants in schools: implications for research and practice	Conference paper	NR	NAO/Humanoid	Body/gesture	Constructs: Engagement; Learning. Methods: Review.
2024	[39]	Social Stories for Promoting Social Communication with Children with Autism Spectrum Disorder Using a Humanoid Robot: Step-by-Step Study	Journal article	Children with ASD	ZECA/Zeno R50/Animal-like/zoomorphic	Face/FER; Body/gesture; Gaze/eye; Task/game performance; Tangible/touch	Constructs: Emotion recognition; Social interaction. Methods: Statistical; Qualitative.
2024	[105]	System Architecture and Hardware Design of a Dog-type Robot for Children Reading Activities	Conference Paper	Children general/non-ASD	NR/Animal-like/zoomorphic	NR	Constructs: NR. Methods: NR.
2024	[93]	Exploring Emotion Classification for Children with Autism in Response to Robot Movement: A Preliminary Case Study in Malaysia	Conference Paper	Children with ASD	NR	NR	Constructs: NR. Methods: ML/DL.
2024	[106]	The Impact of Age and Educational Robotics on Children’s Perception of Robots: A Qualitative Coding Analysis	Conference Paper	Children general/non-ASD	NR	NR	Constructs: Learning. Methods: Qualitative.
2023	[79]	Deep-Learning Based Classification of Engagement for Child-Robot Interaction	Conference paper	Children general/non-ASD	NAO/Humanoid	Face/FER; Body/gesture; Thermal/IR	Constructs: Emotion recognition; Engagement; Learning. Methods: ML/DL.
2023	[107]	Influence of Anthropomorphic Agent on Human Empathy Through Games	Journal article	Children general/non-ASD	NR	Task/game performance; Thermal/IR	Constructs: Stress/arousal; Empathy; Learning. Methods: Statistical.
2023	[41]	Using Physiological Signals and Machine Learning Algorithms to Measure Attentiveness During Robot-Assisted Social Skills Intervention: A Case Study of Two Children with Autism Spectrum Disorder	Journal article	Children with ASD	NAO/Humanoid	Physiology; Task/game performance	Constructs: Social interaction; Learning. Methods: ML/DL; Statistical; Physiological analysis.
2023	[6]	Design Path for a Social Robot for Emotional Communication for Children with Autism Spectrum Disorder (ASD)	Journal article	Children with ASD	Other/Humanoid	Face/FER; Body/gesture; Physiology	Constructs: Emotion recognition; Empathy; Social interaction; Learning. Methods: Qualitative.
2023	[108]	Advanced Metrics to Evaluate Autistic Children’s Attention and Emotions from Facial Characteristics Using a Human-Robot-Game Interface	Conference Paper	Children with ASD	NR	NR	Constructs: Emotion recognition. Methods: NR.
2023	[81]	Measuring Engagement in Robot-Assisted Therapy for Autistic Children	Journal article	Children with ASD	NAO/Humanoid	Face/FER; Body/gesture; Gaze/eye	Constructs: Emotion recognition; Engagement; Stress/arousal; Valence; Social interaction. Methods: ML/DL.
2023	[77]	Do different robot appearances change emotion recognition in children with ASD?	Journal article	Children with ASD	NR	Face/FER; Body/gesture; Gaze/eye; Task/game performance	Constructs: Emotion recognition; Engagement; Social interaction. Methods: ML/DL; Statistical.
2023	[109]	Fully robotic social environment for teaching and practicing affective interaction: Case of teaching emotion recognition skills to children with autism spectrum disorder, a pilot study	Journal article	Children with ASD	Animal-like/zoomorphic	Face/FER; Body/gesture; Task/game performance	Constructs: Emotion recognition; Engagement; Social interaction; Learning. Methods: NR.
2023	[110]	AI-powered backup robot designed to enhance cognitive abilities in children with Down syndrome	Conference paper	Children with ASD	NR	Face/FER; Body/gesture; Voice/audio; Task/game performance	Constructs: Social interaction; Learning. Methods: NR.
2023	[74]	Ethical considerations in child-robot interactions	Journal article	Children with ASD	NR	Body/gesture	Constructs: Empathy. Methods: Review.
2022	[111]	RPPG Detection in Children with Autism Spectrum Disorder during Robot-Child Interaction Studies	Conference Paper	Children with ASD	Other/NR	Face/FER; Body/gesture; Physiology; Thermal/IR	Constructs: Emotion recognition; Social interaction. Methods: Statistical; Wizard-of-Oz; Physiological analysis.
2022	[112]	Towards adaptive and personalized robotic therapy for children with Autism Spectrum Disorder	Conference Paper	Children with ASD	NR	Body/gesture; Physiology; Gaze/eye	Constructs: Engagement; Joint attention. Methods: Physiological analysis; Review.
2022	[113]	Do Robotic Tutors Compromise the Social-Emotional Development of Children?	Journal article	Children general/non-ASD	NAO/Humanoid	Body/gesture	Constructs: Learning. Methods: Qualitative.
2022	[114]	Implementation of Actors’ Emotional Talent into Social Robots Through Capture of Human Head’s Motion and Basic Expression	Journal article	Children with ASD	NAO/Humanoid	Face/FER; Body/gesture; Voice/audio; Gaze/eye	Constructs: Emotion recognition; Engagement; Social interaction; Learning. Methods: NR.
2022	[82]	A Music-Therapy Robotic Platform for Children with Autism: A Pilot Study	Journal article	Children with ASD	NAO/Humanoid	Body/gesture; Physiology; Task/game performance	Constructs: Emotion recognition; Engagement; Stress/arousal. Methods: ML/DL; Physiological analysis.
2022	[43]	Toward Personalized Affect-Aware Socially Assistive Robot Tutors for Long-Term Interventions with Children with Autism	Journal article	Children with ASD	NR	Face/FER; Body/gesture; Voice/audio; Gaze/eye; Task/game performance	Constructs: Engagement; Stress/arousal; Valence; Learning. Methods: ML/DL; Statistical.
2022	[40]	Stress Detection of Children with Autism using Physiological Signals in Kaspar Robot-Based Intervention Studies	Conference paper	Children with ASD	Kaspar/Humanoid	Face/FER; Body/gesture; Voice/audio; Physiology; Gaze/eye	Constructs: Emotion recognition; Engagement; Stress/arousal. Methods: Physiological analysis.
2021	[27]	Embodied Robot Models for Interdisciplinary Emotion Research	Journal article	Children general/non-ASD	NR	Face/FER; Body/gesture; Thermal/IR	Constructs: Emotion recognition. Methods: NR.
2021	[115]	Designing Emotionally Expressive Social Commentary to Facilitate Child-Robot Interaction	Conference paper	Children general/non-ASD	NR	Face/FER; Body/gesture	Constructs: Engagement; Learning. Methods: Qualitative; Wizard-of-Oz.
2021	[116]	A robotic edutainment framework for designing child-robot interaction scenarios	Conference paper	Children general/non-ASD	NAO/Humanoid	Face/FER; Body/gesture; Voice/audio; Task/game performance; Thermal/IR	Constructs: Emotion recognition; Engagement; Empathy. Methods: NR.
2021	[117]	Emotional Expression in Children with ASD: A Pre-Study on a Two-Group Pre-Post-Test Design Comparing Robot-Based and Computer-Based Training	Journal article	Children with ASD	NAO/Humanoid	Face/FER; Body/gesture; Voice/audio	Constructs: Engagement; Social interaction. Methods: Statistical; Qualitative.
2021	[118]	Existing robotics technologies for implementation of special education	Conference Paper	NR	NR	NR	Constructs: Learning. Methods: NR.
2021	[119]	Future Teachers Choose Ideal Characteristics for Robot Peer-Tutor in Real Class Environment	Conference paper	Children with ASD	NAO/Humanoid	Face/FER; Body/gesture	Constructs: Learning. Methods: Statistical.
2021	[39]	Hybrid Approach to Promote Social Interaction with Children with Autism Spectrum Disorder	Conference paper	Children with ASD	ZECA/Zeno R50/Animal-like/zoomorphic	Face/FER; Body/gesture; Gaze/eye; Task/game performance; Tangible/touch	Constructs: Emotion recognition; Engagement; Social interaction. Methods: Statistical.
2020	[84]	Impact of Interaction Context on the Student Affect-Learning Relationship in Child-Robot Interaction	Conference paper	Children general/non-ASD	Tega/NR	Face/FER; Physiology; Task/game performance; Text/LLM; Thermal/IR	Constructs: Emotion recognition; Engagement; Valence; Learning. Methods: Statistical; Physiological analysis.
2020	[46]	Physiological Data-Based Evaluation of a Social Robot Navigation System	Conference paper	No empirical participants/planned	NR	Face/FER; Body/gesture; Voice/audio; Physiology; Thermal/IR	Constructs: Stress/arousal. Methods: ML/DL; Physiological analysis.
2020	[120]	Outcomes of a Robot-Assisted Social-Emotional Understanding Intervention for Young Children with Autism Spectrum Disorders	Journal article	Children with ASD	NAO/Humanoid	Face/FER; Body/gesture; Gaze/eye; Task/game performance; Text/LLM	Constructs: Emotion recognition; Engagement; Learning. Methods: Statistical.
2020	[66]	Communication with self-growing character to develop physically growing robot toy agent	Journal article	Children with ASD	NR/Humanoid	Face/FER; Gaze/eye	Constructs: Emotion recognition; Engagement; Empathy; Social interaction; Learning. Methods: NR.
2020	[45]	Robot Eye Perspective in Perceiving Facial Expressions in Interaction with Children with Autism	Conference paper	Children with ASD	Kaspar/Humanoid	Face/FER; Body/gesture; Gaze/eye; Text/LLM	Constructs: Emotion recognition; Stress/arousal; Valence. Methods: Statistical; Wizard-of-Oz.
2019	[121]	The Role of Trust and Social Behaviours in Children’s Learning from Social Robots	Conference Paper	Children general/non-ASD	NR	NR	Constructs: Learning. Methods: NR.
2019	[33]	Fusing Body Posture with Facial Expressions for Joint Recognition of Affect in Child–Robot Interaction	Journal article	Children general/non-ASD	ZECA/Zeno R50/Humanoid	Face/FER; Body/gesture	Constructs: Emotion recognition; Learning. Methods: ML/DL.
2019	[122]	A Model for an Affective Non-Expensive Utility-Based Decision Agent	Journal article	No empirical participants/planned	NR	Body/gesture; Task/game performance	Constructs: Emotion recognition; Learning. Methods: NR.
2019	[123]	Pomelo, a Collaborative Education Technology Interaction Robot	Conference paper	No empirical participants/planned	NR/Animal-like/zoomorphic	Face/FER; Body/gesture; Voice/audio; Gaze/eye; Task/game performance	Constructs: Emotion recognition; Learning. Methods: NR.
2019	[124]	The Development of Attention Detection Model from Child Behavior for Robot-Assisted Autism Therapy	Conference Paper	Children with ASD	NR	NR	Constructs: NR. Methods: NR.
2019	[125]	Towards Emotional Intelligence in Social Robots Designed for Children	Conference paper	Children general/non-ASD	NAO/Humanoid	Face/FER; Body/gesture; Task/game performance	Constructs: Emotion recognition. Methods: NR.
2019	[47]	Socio-emotional development in high functioning children with Autism Spectrum Disorders using a humanoid robot	Journal Article	Children with ASD	Other/Humanoid	NR	Constructs: NR. Methods: NR.
2019	[126]	Social robot in diagnosis of autism among preschool children	Conference Paper	Children with ASD	NR	NR	Constructs: NR. Methods: NR.
2019	[35]	Affect Recognition in Autism: A single case study on integrating a humanoid robot in a standard therapy	Journal article	Children with ASD	NAO/Humanoid	Face/FER; Body/gesture; Task/game performance	Constructs: Emotion recognition; Engagement. Methods: Wizard-of-Oz.
2018	[127]	Socially-Assistive Robots to Enhance Learning for Secondary Students with Intellectual Disabilities and Autism	Conference Paper	ASD population	NR	Face/FER; Text/LLM	Constructs: Learning. Methods: NR.
2018	[128]	A Pilot Study on Facial Expression Recognition Ability of Autistic Children Using Ryan, A Rear-Projected Humanoid Robot	Conference paper	Children with ASD	NR/Humanoid	Face/FER; Body/gesture	Constructs: Emotion recognition; Empathy; Social interaction. Methods: Statistical.
2018	[11]	Personalized machine learning for robot perception of affect and engagement in autism therapy	Journal article	Children with ASD	NAO/Humanoid	Face/FER; Body/gesture; Voice/audio; Physiology; Text/LLM	Constructs: Emotion recognition; Engagement; Stress/arousal; Valence; Learning. Methods: ML/DL; Physiological analysis.
2018	[129]	Can Dogs Assist Children with Severe Autism Spectrum Disorder in Complying with Challenging Demands? An Exploratory Experiment with a Live and a Robotic Dog	Conference Paper	Children with ASD	Unitree Go2 robotic dog/Animal-like/zoomorphic	NR.	Constructs: NR. Methods: NR.
2018	[34]	3D Human Sensing, Action and Emotion Recognition in Robot Assisted Therapy of Children with Autism	Conference paper	Children with ASD	NR/Humanoid	Face/FER; Body/gesture; Text/LLM; Thermal/IR	Constructs: Emotion recognition; Stress/arousal; Valence; Learning. Methods: ML/DL.
2018	[130]	Analyzing children’s affective reactions and preferences towards social robots using paralinguistic and self-reported information	Conference Paper	Children general/non-ASD	NR	NR.	Constructs: NR. Methods: NR.
2018	[129]	Building a hybrid approach for a game scenario using a tangible interface in human robot interaction	Conference Paper	Not specified	NR/Hybrid with tangible interface	NR	Constructs: NR. Methods: NR.
2017	[131]	Improving therapeutic outcomes in autism spectrum disorders: Enhancing social communication and sensory processing through the use of interactive robots	Journal article	Children with ASD	NAO/Animal-like/zoomorphic	Face/FER; Body/gesture; Voice/audio; Gaze/eye	Constructs: Engagement; Social interaction; Joint attention. Methods: Review.
2017	[78]	Detecting perceived quality of interaction with a robot using contextual features	Journal article	Children with ASD	NR	Gaze/eye; Task/game performance; Text/LLM	Constructs: Emotion recognition; Engagement; Valence; Empathy; Learning. Methods: ML/DL.
2017	[132]	Social skills training for children with autism spectrum disorder using a robotic behavioral intervention system	Journal Article	Children with ASD	NR	NR	Constructs: Social interaction. Methods: NR.
2017	[133]	A Feasibility Study Evaluating the Emotionally Expressive Robot SAM	Journal article	Children with ASD	NR/Animal-like/zoomorphic	Face/FER; Body/gesture; Gaze/eye; Thermal/IR	Constructs: Emotion recognition; Engagement; Social interaction. Methods: Statistical.
2017	[134]	Cloud-assisted hugtive robot for affective interaction	Journal article	Children with ASD	NR	Body/gesture; Physiology; Task/game performance; Text/LLM	Constructs: Emotion recognition; Stress/arousal; Valence; Social interaction. Methods: ML/DL; Wizard-of-Oz; Physiological analysis.
2017	[44]	Robot-assisted therapy for learning and social interaction of children with autism spectrum disorder	Journal article	Children with ASD	KiliRo/Animal-like/zoomorphic	Face/FER; Body/gesture	Constructs: Emotion recognition; Social interaction; Learning. Methods: Qualitative.
2017	[36]	Measuring engagement in robot-assisted autism Therapy: A crosscultural study	Journal article	Children with ASD	NAO/Humanoid	Face/FER; Task/game performance; Text/LLM	Constructs: Engagement; Stress/arousal; Valence. Methods: Statistical.
2016	[44]	Experimental evaluation of parrot-inspired robot and adapted model-rival method for teaching children with autism	Conference paper	Children with ASD	KiliRo/Animal-like/zoomorphic	Face/FER; Body/gesture	Constructs: Emotion recognition; Social interaction; Learning. Methods: ML/DL; Statistical; Wizard-of-Oz.
2016	[135]	Emotional Robot to Examine Different Play Patterns and Affective Responses of Children with and without ASD	Conference paper	Children with ASD	NR	Face/FER; Task/game performance; Tangible/touch	Constructs: Empathy. Methods: Statistical.
2016	[136]	Ethically-Guided Emotional Responses for Social Robots: Should I Be Angry?	Conference paper	No empirical participants/planned	NR	Body/gesture	Constructs: NR. Methods: NR.
2016	[137]	Give Children Toys Robots to Educate and/or Neuroreeducate: The Example of PEKOPPA	Conference Paper	Children general/non-ASD	Pekoppa/NR	NR	Constructs: NR. Methods: NR.
2016	[38]	A multimodal and multilevel system for robotics treatment of autism in children	Conference paper	Children with ASD	ZECA/Zeno R50/Humanoid	Face/FER; Body/gesture; Gaze/eye	Constructs: Emotion recognition; Joint attention. Methods: Statistical.
2016	[138]	It’s All in the Eyes: Designing Facial Expressions for an Interactive Robot Therapy Coach for Children	Conference Paper	Children general/non-ASD	NR	NR	Constructs: Emotion recognition. Methods: NR.
2015	[139]	Potential clinical impact of positive affect in robot interactions for autism intervention	Conference paper	Children with ASD	Pleo/Animal-like/zoomorphic	Task/game performance	Constructs: Engagement; Valence; Social interaction. Methods: Statistical; Wizard-of-Oz.
2015	[140]	Autonomously detecting interaction with an affective robot to explore connection to developmental ability	Conference paper	Children general/non-ASD	Other/Humanoid	Face/FER; Body/gesture; Tangible/touch; Text/LLM	Constructs: Emotion recognition; Engagement; Valence. Methods: Statistical.
2015	[80]	Automatic Emotion Recognition in Robot-Children Interaction for ASD Treatment	Conference paper	Children with ASD	NR	Face/FER; Body/gesture	Constructs: Emotion recognition; Social interaction; Learning. Methods: ML/DL.
2015	[141]	An emotion recognition comparative study of autistic and typically-developing children using the zeno robot	Conference Paper	Children with ASD	ZECA/Zeno R50/Humanoid	Face/FER; Body/gesture; Task/game performance	Constructs: Emotion recognition. Methods: Statistical.
2015	[142]	Engagement: A traceable motivational concept in human-robot interaction	Conference paper	No empirical participants/planned	NR	Voice/audio; Gaze/eye; Task/game performance; Thermal/IR	Constructs: Engagement; Social interaction. Methods: NR.
2015	[141]	An Emotion Recognition Comparative Study of Autistic and Typically-Developing Children using the Zeno Robot	Conference Paper	Children with ASD	ZECA/Zeno R50/Humanoid	NR	Constructs: Emotion recognition. Methods: NR.
2014	[58]	Adaptive Emotional Expression in Robot-Child Interaction	Conference paper	Children general/non-ASD	NAO/Humanoid	Face/FER; Body/gesture; Voice/audio; Gaze/eye; Task/game performance; Thermal/IR	Constructs: Emotion recognition; Engagement; Stress/arousal; Valence; Empathy. Methods: Wizard-of-Oz.
2014	[143]	Building a game scenario to encourage children with autism to recognize and label emotions using a humanoid robot	Conference paper	Children with ASD	ZECA/Zeno R50/Humanoid	Face/FER; Body/gesture; Task/game performance; Thermal/IR	Constructs: Emotion recognition; Engagement; Learning. Methods: NR.
2014	[144]	A pilot study using imitation and storytelling scenarios as activities for labelling emotions by children with autism using a humanoid robot	Conference paper	Children with ASD	ZECA/Zeno R50/Humanoid	Face/FER; Body/gesture; Physiology; Task/game performance; Thermal/IR	Constructs: Emotion recognition; Engagement; Empathy; Social interaction. Methods: Qualitative.
2014	[145]	The MEI Robot: Towards Using Motherese to Develop Multimodal Emotional Intelligence	Journal article	No empirical participants/planned	NR	Face/FER; Body/gesture; Voice/audio	Constructs: Emotion recognition. Methods: NR.
2014	[146]	Emotional Interaction with a Mobile Robot using Hand Gestures	Conference paper	No empirical participants/planned	Non-humanoid/mobile/manipulator	Body/gesture	Constructs: Emotion recognition. Methods: NR.
2014	[147]	Child’s Perception of Robot’s Emotions: Effects of Platform, Context and Experience	Journal article	Children general/non-ASD	NAO/Humanoid	Face/FER; Body/gesture; Text/LLM	Constructs: Emotion recognition; Learning. Methods: Statistical.
2014	[148]	Neurotypical and autistic children aged 6 to 7 years in a speaker-listener situation with a human or a minimalist InterActor robot	Conference paper	Children with ASD	Pekoppa/non-Humanoid	Face/FER; Body/gesture; Physiology	Constructs: Engagement; Social interaction. Methods: Statistical.

*Note.* The table reports the 105 studies included in the final corpus, ordered by publication year from newest to oldest. Categories for sensing features, constructs, and methods are not mutually exclusive because a study may include multiple modalities or approaches. “NR” indicates that the information was not reported or could not be determined with sufficient confidence from the original article. “NA” indicates that the variable was not applicable to the study design or analytical approach. “Other” indicates explicitly reported information that did not fit the predefined coding categories. Missing values were not imputed.
